# Temperature-Driven Biodiversity Change: Disentangling Space and Time

**DOI:** 10.1093/biosci/biy096

**Published:** 2018-09-19

**Authors:** Conor Waldock, Maria Dornelas, Amanda E Bates

**Affiliations:** 1 Ecological impacts of climate warming at the University of Southampton under the supervision of Amanda E. Bates; 2 Associate professor and Canada research chair in marine physiological ecology at Memorial University of Newfoundland; 3 Maria Dornelas, reader at The University of St Andrews, is a macroecologist focused on biodiversity patterns

**Keywords:** climate change, climate velocity, community dynamics, species redistribution, range shifts

## Abstract

Temperature regimes have multiple spatial and temporal dimensions that have different impacts on biodiversity. Signatures of warming across these dimensions may contribute uniquely to the large-scale species redistributions and abundance changes that underpin community dynamics. A comprehensive review of the literature reveals that 86% of studies were focused on community responses to temperature aggregated over spatial or temporal dimensions (e.g., mean, median, or extremes). Therefore, the effects of temperature variation in space and time on biodiversity remain generally unquantified. In the present article, we argue that this focus on aggregated temperature measures may limit advancing our understanding of how communities are being altered by climate change. In light of this, we map the cause-and-effect pathways between the different dimensions of temperature change and communities in space and time. A broadened focus, shifted toward a multidimensional perspective of temperature, will allow better interpretation and prediction of biodiversity change and more robust management and conservation strategies.

Environmental temperature is a primary variable important for biological function at all organizational scales. Even slight temperature changes can dramatically affect biological processes from cells to populations, with strong ecological consequences. At the smallest scale, temperature drives cellular reaction rates through kinetic processes. Individuals respond directly to environmental temperature—for example, by modulating their activity rates (Payne et al. 2016, Payne 2018). Population demographic rates are also temperature sensitive (Dell et al. 2011), with consequences for abundance and occupancy patterns. Demographic changes in combination with individual effects (e.g., foraging velocity, ingestion rates; Dell et al. 2011) lead to shifts in species interaction strengths (disease prevalence, Kock et al. 2018; parasitism rate, Runjie et al. 1996), ultimately translating to change in community dynamics and structure (Kordas et al. 2011, Bellard et al. 2012).

Environmental temperature is highly variable in both space and time. Some aspects of environmental temperature are predictable (e.g., seasonal changes), others are not (e.g., extreme events). Therefore, a *temperature regime* has multiple dimensions that can be described in both space and time, with the potential to shape biological patterns in different ways (Garcia et al. 2014). For example, as we move from the tropics to the poles, it becomes colder, daily variability decreases, but seasonal variability increases (Wang and Dillon 2014). Distinct signatures of spatial variability also exist; for example, temperature is much more spatially heterogeneous in intertidal systems than in subtidal systems. Along these different axes of temperature dimensions in space and time, there is an additional layer of gradual long-term warming due to anthropogenic climate forcing (IPCC 2013).

Climate change is altering environmental temperature regimes. The spatial arrangement (i.e., spatial heterogeneity) and timing of temperature change is typically abstracted to a statistical distribution, defined presently and by others as *temperature magnitude* (see box 1 and Garcia et al. 2014). However*,* by examining only temperature magnitude (e.g., its mean), we are deprived of the detailed dynamics of spatial and temporal temperature change. The *position* and *availability* dimensions of temperature change can vary, even without shifts in the overall mean temperature of a region or through time (see box 1 and figure 1 for further definitions; Garcia et al. 2014). For example, the movement of thermal isoclines with warming is an example of a shift in the *position* of temperature in space (Loarie et al. 2009, Burrows et al. 2011, Hamann et al. 2015). The earlier onset of spring represents a change in *position* of temperature in time (Wang and Dillon 2014). Changes to the *availability* of temperature depend on physical area or temporal duration of particular temperature signatures within a region or time slice (Williams et al. 2007, Ordonez and Williams 2013).

**Figure 1.**
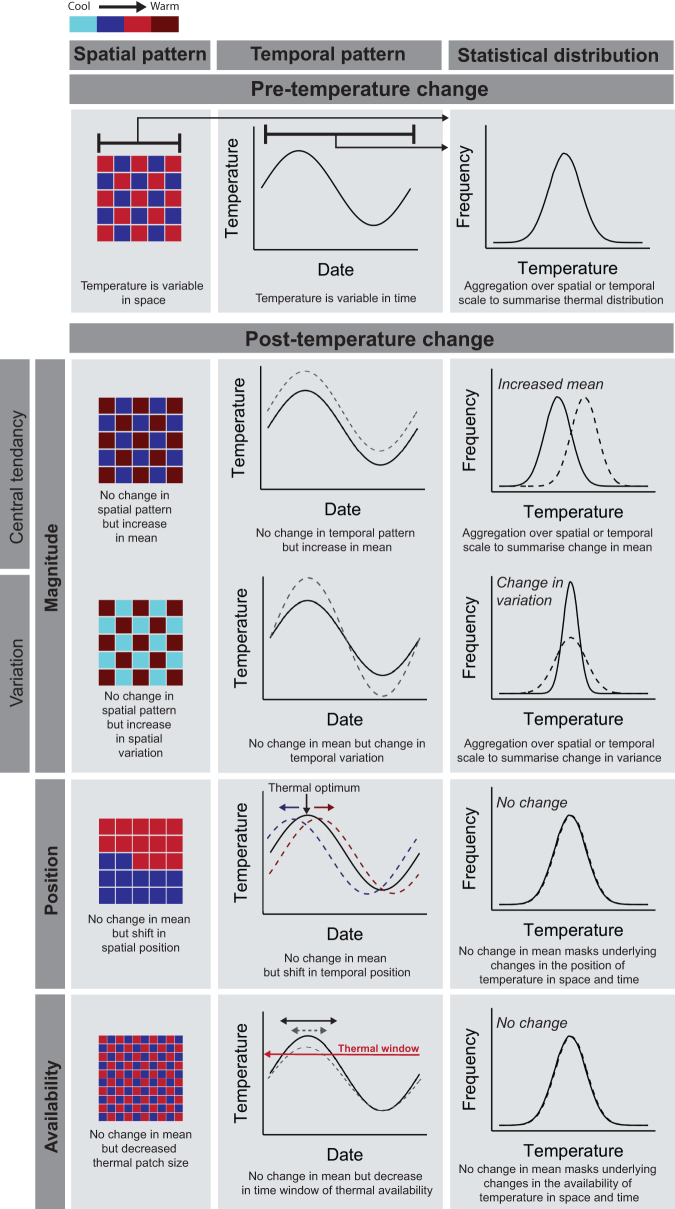
Temperature patterns in defined spatial and temporal units and their change through time. Temperature is variable in both space and time, but to aggregate to a mean value, a researcher must select scales of space and time to describe a particular statistical temperature distribution. Changes in a statistical distribution (e.g., central tendency and variation) can occur with no changes to the underlying spatial or temporal organization of temperature within those defined units (e.g., 50 square kilometers, 1 year). Likewise, even if no change is observed in the statistical distribution, underlying shifts in space and time may be masked, in both availability and position of temperature. We outline a few, of many, possible scenarios by which temperature magnitude, position, and availability can change.

**Box 1. Defining and measuring temperature and biodiversity change in space and time.**

Temperature regimes—the characteristic pattern of temperature variation in space and time for a given scale—vary across the Earth. For example, temperate regions since the Late Quaternary have been characterized by cool and warm periods (i.e., seasonality) through the year and relative cool climates. Four different dimensions of a temperature regime are generally recognized (see Garcia et al. 2014), and how they vary with climate change is described in the present article (see figure 1). *Temperature magnitude* describes the change in the statistical distribution of temperature for a given locality. The statistical distribution is defined by both its central tendency (e.g., changing mean—i.e., warming or cooling) and its dispersion (e.g., increasing variation, skew or kurtosis—i.e., extreme events). The rate of change in temperature magnitude is defined by the change in the statistical parameter (e.g., mean, standard deviation) per unit time.

Underlying this change in temperature magnitude are changes to temperature position and temperature availability in space and time (figure 1). *Temperature temporal position* describes the change in the timing (i.e., date) of a specific temperature event within a defined spatial unit. This contrasts with *temperature temporal availability*, which describes a change in the total duration of a specific temperature event within a defined spatial unit. *Temperature spatial position* is defined by the relocation of temperature to a new area (i.e., isotherm shift change in linear distance) for a given temporal unit (Loarie et al. 2009, Hamann et al. 2015, figure 3). In contrast, *temperature spatial availability* measures the change in area or size of a temperature available within a geographic location (i.e., the change in geographic space of a temperature regime measured in square kilometers, km^2^) for a given temporal unit (figure 3). Novel climates are an important component of temperature spatial availability and describe the availability of new climatic space, increasing from an initial area of 0 km^2^ (Williams and Jackson 2007).

Likewise, community metrics also fall into the following broad categories to measure structural and compositional differences in species assembled at local scales: species richness, total abundance, species relative abundance, compositional and trait based (Smith et al. 2009, Magurran and McGill 2011, Hill et al. 2016, Santini et al. 2016). These are important to recognize when matching the measured community responses to the processes driving change (i.e., “Linking community change processes and temperature dimensions”). The total number of different species in a community is measured using species richness metrics. The net loss and gain of species translates to a change in richness. The total abundance of a community is simply the sum of all individuals in a community and is often related to species richness as a result of sampling effects (i.e., more individuals increases the probability of a new species being present). The distribution of individuals between species represents the structure of a community and is often summarized by the shape of species relative abundance distributions. A change in structure occurs with shifts in species relative abundances (e.g., few rare species versus many common species), but these changes are agnostic to species identity (i.e., the same structure, but the assemblage comprises all new species). Therefore, structural change can represent richness and total abundance changes simultaneously. Compositional metrics describe how both species’ relative abundances and identities shift and therefore measure the reorganization of species abundances in a community. The losses or gains of species measures the turnover component of compositional change (Baselga and Leprieur 2015). Finally, trait-based metrics quantify the diversity, range and values of the traits and niche properties of species within a community; these are often relevant to a particular driver of interest (e.g., species thermal limits and warming).

In the present article, we identify the underlying spatial and temporal components (availability and position) overlooked by summary distributions (magnitude). For example, as isotherms shift away from a particular location, warming at the location may occur, but the spatial context of temperature change will influence the regional setting of biodiversity change. Changes to the spatial and temporal arrangement and location of temperatures can often be statistically independent of mean temperature changes and, therefore, decoupled from mean temperature changes (Garcia et al. 2014). Therefore, it is important to ask what we might miss by interpreting biodiversity responses to climate change exclusively as a summary of a statistical distribution and what can be gained by explicitly considering how temperature change manifests in space and time as availability and position change.

There is a further challenge in linking community-level change directly to the different dimensions of temperature change, because this requires disaggregation of a community into meaningful response units. Communities—groups of species that share environments at a given time and location with the potential for species to interact (Fauth et al. 1996)—are complex biological units. As with temperature regimes, communities can also be characterised by many distinct dimensions (see box 1 for definitions). Examples of these dimensions include the number of species (species richness), the total number of individuals (abundance), the distribution of individuals among different species (relative abundance), the combined mass of all individuals (biomass, a measure of energetic consumption and productivity), and the variety of individuals and species (genetic and phylogenetic diversity) and their characteristics (functional diversity). Therefore, different aspects of biodiversity are affected by a suite of factors, leading to difficulty in identifying with confidence which factors are mechanistic drivers of emergent patterns (Lawton 1999). As with considering changes in the temperature regime, the challenge becomes even greater when the additional complexity of community dynamics through time is of interest (McGill et al. 2015). Certain community dimensions are responsive to environmental changes, such as the composition and relative abundance of assemblages (Hill et al. 2016), whereas others, such as species richness, are less directly responsive to environmental change (Santini et al. 2016), with increases or decreases being highly dependent on measurement scale (Vellend et al. 2017). The multidimensional nature of both temperature and biodiversity variables justifies the aim of the present article: to map predictions of cause-and-effect among different dimensions.

## Literature review

**Figure 2.**
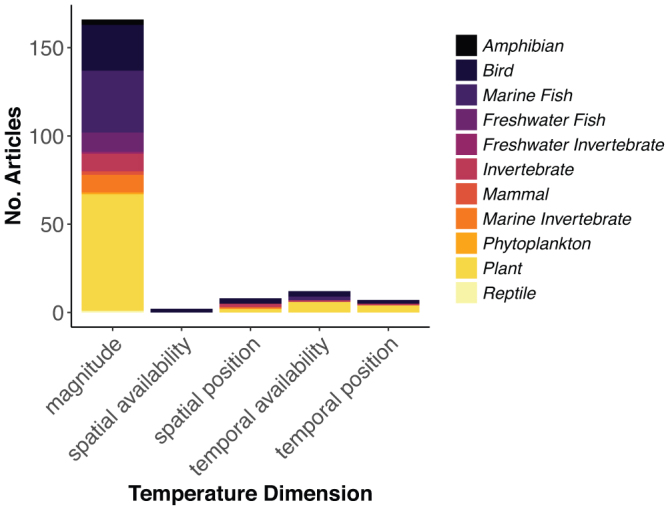
Magnitude of temperature change dominates our understanding of temperature change. The number of articles assessing community response to temperature change per dimension of temperature change representing 11 taxonomic groups from 2005 until 2015.

To determine what dimensions of temperature regime and community responses are most commonly studied, we reviewed articles published from 2005 until 2015 (see the supplemental materials for methods and discussion). We found that, in spite of the complexity in changing temperature regime, most research has been focused on identifying responses to mean warming trends. Of the 156 papers returned from our literature review, 86% were focused on temperature magnitude, and only a small proportion of research has investigated spatial position (3%) and availability (1%) or temporal position (4%) and availability (6%, figure 2). Of the metrics used to describe changes in temperature magnitude—the changing statistical distribution of temperature—mean changes were investigated in 48% of the studies, and 41% of the studies were focused on minimum or maximum temperatures. We also found a strong bias toward species richness (36%) rather than toward species identity (13%) or relative abundance (6%) as the predominant dimension of communities measured in responses to temperature change. We therefore reveal that the spatial and temporal complexity underlying temperature change is rarely considered as a driver of community dynamics, instead temperature is generally abstracted over spatial or temporal scales to an aggregate mean value.

## Objectives and purpose

Trends in the responses of biodiversity that have been detected and attributed to particular dimensions of temperature change in space and time are emerging. No-analogue communities have formed as species reshuffle in response to the development of novel temperature regimes (Urban et al. 2012). Increases and decreases in species richness have been attributed to changing species distributions following locational shifts in thermal isoclines (Devictor et al. 2012, Batt et al. 2017). Changing temperature regimes have also been implicated in driving the increasing relative abundance of widespread and common species, or homogenization, of communities (Davey et al. 2012, Magurran et al. 2015). There is widespread evidence of shifts toward species with thermal preferences for warmer environments (Bates et al. 2014a, Horta e Costa et al. 2014, Gaüzère et al. 2015, Tayleur et al. 2015, Stephens et al. 2016), a process known as *tropicalization* or *thermophilization*.

Notwithstanding these few examples, how different dimensions of temperature change will affect the dynamics of multidimensional communities is poorly established at present. To address this gap, we developed a conceptual framework to guide predictions and explicit quantitative tests of biodiversity change in response to temperature change, measured in the appropriate dimensions of space and time (O’Connor et al. 2015, Houlahan et al. 2017). We further illustrate below why neglecting the dimensionality inherent to both temperature regime and biodiversity change may prevent accurate predictions. We focused on changes in the richness of local communities and homogenization over space as illustrative examples of biodiversity change that are driven by multiple temperature change dimensions.

## Linking community change processes and temperature dimensions

Two community processes are relevant to understand community dynamics at anthropogenic time scales—*selection* and *movement* (Vellend 2010, 2016). The selection and movement of individuals lead to change in richness, structure, composition of communities, and traits of the species present. These processes underpin local community dynamics that form an important component of biodiversity change (e.g., figure 4; Vellend 2010, 2016).

Abundance-related metrics (composition, total and relative abundance; see box 1) are expected to be most responsive to changes in selection processes acting on communities. For example, under changing environmental conditions, deterministic fitness differences between individuals within a population alter birth and death rates (i.e., demographic effects). This has consequences for population dynamics, and populations within a community increase or decrease in abundance. When species differ in the effect of these selection-based changes, variation in population dynamics between different species within a local community occurs. These population changes manifest themselves as changes in composition and relative abundance.

In contrast to selection processes relating to abundance metrics, changes in species richness and identity are expected to be sensitive to environmental changes that alter movement community processes: The immigration or emigration of individuals (either active or passive—e.g., migration or dispersal) into or out of a local community adds or removes species from a community. Most examples of movement mediated richness change come from colonizations of novel species on islands (i.e., MacArthur and Wilson 1967), many of which are driven by human actions in recent times (Sax and Gaines 2008, Vellend et al. 2017). Furthermore, the capacity for species to disperse into communities as they assemble affects climax or equilibrium community richness (Lichter 2000, Makoto and Wilson 2016). Batt and colleagues (2017) reported a novel example from marine benthic fish assemblages in which increasing range size of rare and transient species, through movements to new localities, increased the species richness of any given location within a region.

Movement can also cause additional selection processes by creating interactions between arriving species and those present in the local assemblage and therefore drive additional community changes (Gilman et al. 2010, Urban et al. 2012, Alexander et al. 2015, Vellend 2016). For instance, Alexander and colleagues (2015) found that transplanting competitors into Swiss Alpine plant communities had large effects on the survival, biomass, and flower probability in species of the local assemblage.

### How do community processes respond to specific dimensions of temperature change?

The effects of a change in temperature magnitude (central tendency and variability) on local communities influence the selection process through population birth and death rates, leading to changes in the relative abundance of species found in a community (figure 4). Temperature-related magnitude changes occur through environmental filtering: selection of individuals with higher relative fitness and selection against individuals with low fitness. Changes in temperature magnitude predict biodiversity change, and this, in part, explains why this approach is so commonly adopted. A proximate cause of these community responses is that species often evolve to optimize temperatures frequently experienced, leading to a peak in performance (Angilletta 2009). In addition, temperature variability can exceed species’ limits (e.g., Mckechnie and Wolf 2010, Dowd et al. 2015). For example, modeled population trends of water and sea birds across the United Kingdom, based partly on summer and winter temperature extremes, predict 56% of variation in average the population dynamics of birds in this region (Johnston et al. 2013; see the supplemental materials for further discussion).

**Figure 3.**
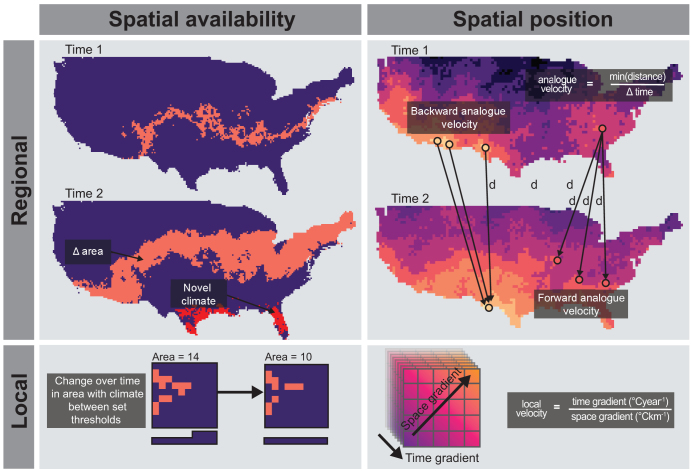
Schematic diagram of spatial dimensions of temperature change. Spatial availability is represented in the present article as differences in the total geographic area of thermal niche at a regional scale. Novel climates may emerge, representing newly available temperature regimes. Spatial position can be measured as forward or backward temperature velocity, which are proxies for different ecological processes of emigration and immigration respectively (Carroll et al. 2015, Hamann et al. 2015). Analogue velocity is the minimum distance necessary to travel to maintain constant temperature conditions (Hamann et al. 2015). Local climate velocity is the rate of temporal change in temperature over the spatial gradient of temperature (Loarie et al. 2009). Adapted from Garcia and colleagues (2014) and Carroll and colleagues (2015).

**Figure 4.**
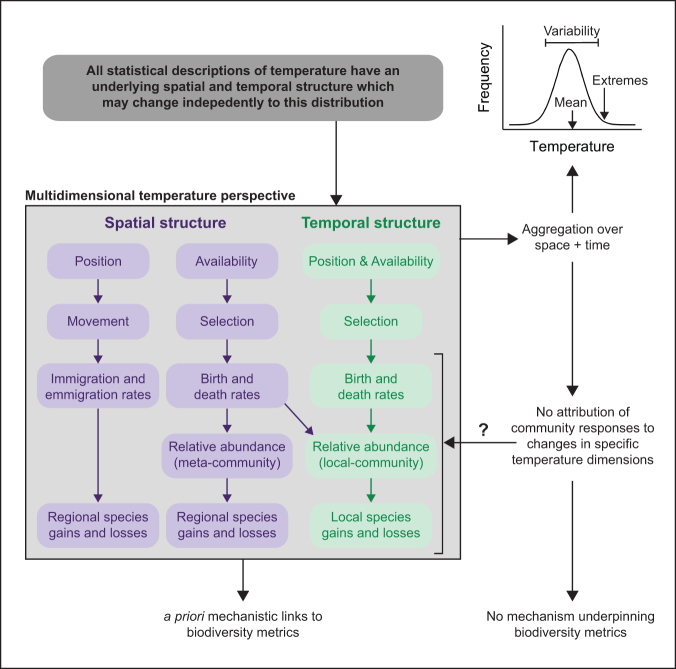
Framework linking changing temperature dimensions to processes that drive community level responses. Applying a multidimensional perspective explicitly accounts for temperature changes in space and time that affect biodiversity that occurs through selection and movement pathways. Population-level effects arise when selection influences birth and growth rates, and movement influences immigration and emigration rates. Changes in population and demographic rates drive changes in the relative abundances and richness of regional and local communities. If we aggregate temperature over space and time, we miss the opportunity to attribute community responses to changes in specific temperature dimensions. We also lack the resolution to build a priori hypotheses with mechanistic links between changes in the physical environment and individuals’ responses.

However, complexities of temperature change in space and time are missed when considering temperature exclusively from this perspective. How the selection and movement of individuals respond to changes in temperature dimensions depends on the spatial and temporal nature of temperature changes (figures 1, 3). Considering the dimensionality of temperature leads to different predictions for how biodiversity will change with warming (Garcia et al. 2014, Ordonez et al. 2013, described in figure 4).

### Local scale shifts in temporal position and availability

The temporal position and availability dimensions of temperature change affect local community selection processes. Shifts in temporal position (e.g., seasonality and the earlier onset of spring, figure 1) and temporal availability (e.g., duration of temperatures above a physiological threshold, figure 1) drive demographic change (Jones and Wiman 2012, Gaillard et al. 2013, Matechou et al. 2014, figure 4). For instance, variation in spring timing (temporal position) between years reduced roe deer (*Capreolus capreolus*) population growth rates by limiting successful spring recruitment (Gaillard et al. 2013). In another example, broods in common blue butterfly (*Polyommatus carus*) populations emerged later at higher latitudes, because of differences in the availability of spring and summer temperatures. This change in temporal availability constrained the total broods within the year at northern sites, leading to a smaller overall population size (Matechou et al. 2014).

In ecosystems with seasonal cycles in temperature, a key indirect driver of community change is the mismatch in timing of life history events for species with strong dependencies. This effect is exacerbated if entire groups of species that interact have different capacities to respond to temporal position or, if changes to temporal availability alter how life-history stages transition (i.e., development times). Compelling examples are known of mismatches among resources, consumers, and predators. In a now classic example, Both and colleagues (2009) found that, for passerine birds, climate change led to advances in temporal position of caterpillar prey peak abundance that were unmatched by changes in peak food demand. The predators of these passerine birds did not shift the date of their energy requirements to keep pace with changing prey fledgling availability, and therefore, mismatches occurred at multiple levels across an ecological assemblage (Both et al. 2009). Similar mismatches in key seasonal timings across trophic levels were found with climate change for 726 plant, vertebrate and invertebrate taxa in the United Kingdom (Thackeray et al. 2010). Large-scale compositional changes are expected to occur in communities undergoing mismatches in the timings of species present, because mismatches lead to performance (and abundance) declines of species lacking the resources required within in a specific time window.

## Change in spatial dimensions of temperature change through time

The distance between habitat patches and habitat area are two key components of spatially explicit ecological theories, such as metapopulation patch dynamics (Hanski 1998) and island biogeography (MacArthur and Wilson 1967). We suggest that parallels can be drawn for spatial temperature dimensions. For example, temperature availability relates to patch or island size, and temperature position relates to interpatch distances or island distance from a mainland. We use analogous ideas to explore the changing position and availability of temperature in generating community dynamics.

### Spatial availability

Temperature spatial availability measures the geographic area of temperature (i.e., spatial extent) within species’ niche limits (figure 3). The effects of changing temperature spatial availability are dependent on scale. At a local scale, selection processes in communities determine change because temperature availability at a local scale can be viewed as an ecological resource (Magnuson et al. 1979, Roughgarden et al. 1981) for which individuals compete (Melville 2002). Therefore, individuals’ performance and population abundance can be affected by changing geographic area of thermal resources and habitat patches (Matthiopoulos et al. 2015). For example, fragmentation of primary forests leads to patches of matrix that can be many degrees warmer than contiguous forest (Senior et al. 2017), and the size of these warm patches is expected to influence the space use, behavior, and survival of populations of species dependent on forest habitats (Tuff et al. 2016).

At regional to global spatial scales, the available area of thermal niche limits species geographic range sizes and, therefore, a species’ global abundance as the two are strongly linked (Borregaard and Rahbek 2010). Limited availability of areas within the limits of the thermal niche leads to an increased probability of extinctions if populations shrink in geographic area and abundance (Purvis et al. 2000). As such, for a regional community, selection processes are important because the geographic extent of a preferred climate directly constrains species range extents, which deterministically affects species abundances. In the Pleistocene, a period of rapid temperature changes, species’ extinctions occurred at higher rates in regions in which climate refugia were not sufficiently large to maintain viable populations (e.g., Hofreiter and Stewart 2009, Nogues-Bravo et al. 2010). In this period, species with large body sizes were particularly sensitive to temperature availability change because of low density and large ranges (Lyons et al. 2004, Barnosky 2008). The polar bear (*Ursus maritimus*) provides a modern analogue of a species with increased risk of population extinctions due to spatial availability of temperature-dependent habitat. For this species, there is a predicted 68% reduction in summer habitat availability by the end of the century (Durner et al. 2009).

**Figure 5.**
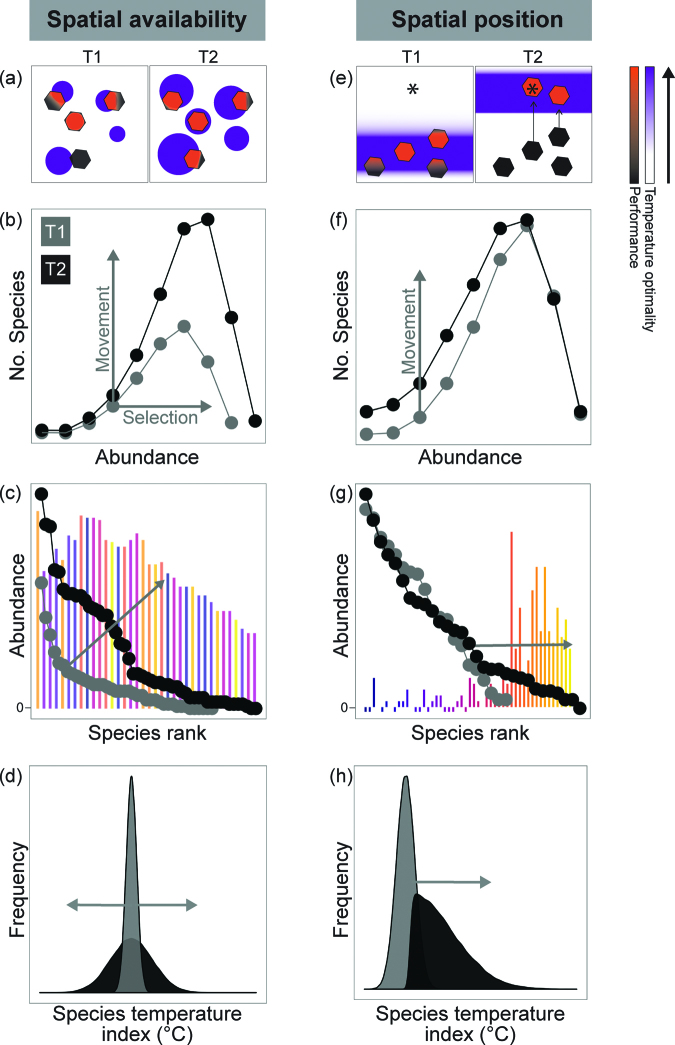
Schematic diagram linking changes in spatial availability and position of temperature with community responses using different biodiversity metrics. In panels (a) and (e), the polygons represent individuals, and the purple (dark) regions represent areas of temperature optimality. In panels (b) and (f), species abundance distributions show a right shift in central tendency with increased abundance, an increase in height with increasing richness. In panesl (c) and (g), rank abundance distributions show an increasing tail with higher richness and a shift right with increasing total abundance. Note the long tail for temperature position change. The underlying bars represent species abundance change between time points but maintained at the rank on the x-axis in time point 1. The colors refer to thermal traits (blue (dark) is cool affinity; yellow (light) is warm affinity). In panels (d) and (h), changes in the distribution of species’ thermal affinities between time points is the mean of this distribution. Note the same mean for changes in spatial availability and the long tail and changing mean for spatial position.

The spatial context of changes in temperature availability, rather than aggregated temperature data alone, provides additional insights to community responses to temperature change. As one example, if there is greater geographic area of temperature available, at either local or regional scales, we expect populations in environments of more optimal temperatures to increase in size, potentially increasing the total abundance of a community too (Cline et al. 2013). This prediction requires testing in model systems that disentangle the area of temperature availability from habitat size more generally. Figure 5 provides a visual representation of multiple dimensions of community responses, using species- and rank-abundance distributions and community temperature index. These predictions are in contrast with mean temperature change, which predicts that different species may decrease or increase in abundance depending on which thermal habitats are preferred by individuals, such that total abundance may not increase (locally or regionally; Johnston et al. 2013). Sampling more individuals (with increased temperature availability) increases variation of community traits from sampling effects alone (i.e., increase variation in species thermal affinities but no directional shift in community average thermal affinity as would occur with mean warming). Richness will increase if movements into communities are concurrent with greater area of temperature available, through species-area effects (Brose et al. 2004).

A special case of spatial availability change is the emergence of a novel climate (Williams et al. 2007, Ordonez and Williams 2013). Novel climates can be considered new temperature regimes that were globally unavailable during the evolution of species in the regional fauna, as have occurred frequently throughout Earth's geological history. Given that the size of available climates is limited by the size of the planet, an area of novel climate space must exclude or replace an area of present day climate space. Within novel climate space, no-analogue species assemblages are expected to form with corresponding shifts in species composition through time because of interspecific differences in climate tolerances (Williams and Jackson 2007). The ecological implications of emerging novel climates are extremely difficult to anticipate, and ecological surprises are expected to unfold with new species interacting for the first time (Radeloff et al. 2015).

### Spatial position

A change in temperature spatial position measures the geographic distance a specific temperature (thermal isocline) shifts after a climatic change (i.e., spatial distance in kilometers). Temperature velocity, the rate of spatial shift in thermal isoclines (figure 3; measured as the rate per km per decade), is a frequently used metric to measure changes in the spatial position (Loarie et al. 2009, Hamann et al. 2015). Therefore, the movement of individuals is an important mechanism underpinning community responses to this temperature dimension. For example, individual leatherback turtle (*Dermochelys coriacea*) movements tracked 15-degree-Celsius isotherms (McMahon and Hays 2006), and shifts in species ranges are also well documented and are increasingly predicted to track the position of preferred temperatures with warming (Devictor et al. 2008, 2012, Burrows et al. 2011, Pinsky et al. 2013, Hiddink et al. 2015, Sunday et al. 2015). Pinsky and colleagues (2013) demonstrated that range centroids have tracked the position of thermal isoclines with climate variability in the last 50 years in 360 marine taxa.

Identifying whether individuals move within or outside of their geographic range is also important when interpreting biodiversity change (Lenoir and Svenning 2015). Movement outside of a geographic range—that is, colonization of a new habitat—will result in species richness and species identity change for a receiving community (see the tail of rank-abundance distributions in figure 5g). These initially rare species may become more common over longer temporal scales because of increases in population sizes (in figure 5g, the rare species shift leftward in rank abundance distributions). Conversely, shifts in spatial position can lead to richness declines when extirpations of individuals emigrate from communities, which result in local absences (independent of selection processes altering birth and death rates). The direction of the richness change depends on the relative positioning of species’ range edges across the community. Communities receive species that are on a leading range edge but lose species at a contracting range edge. Shifting isotherms may also elicit species relocations within ranges, and therefore, the relative abundances of species is expected shift to follow these isotherms. This process could act independently of local abundance change driven by a change in temperature magnitude and selection processes (figure 4).

Species traits cause variation in individuals’ response to the position dimension of temperature change, implicating the importance of trait-based metrics (e.g., Sunday et al. 2015). For example, at the community level the average species’ thermal affinity in a community, often summarized as a community temperature index, is expected to be sensitive to the spatial position dimension of temperature change (figure 4). With the establishment of warmer tolerant colonists, we expect the community temperature index to increase and the distribution of species’ thermal affinities to become increasingly right skewed (figure 5h; ter Hofstede et al. 2010, Bates et al. 2014b). This is in contrast to predictions from changes in temperature availability, according to which only increased variation but no mean change in species thermal affinities is expected (figure 5d cf. 5h). Furthermore, species with high mobility have better capacity to keep pace with spatial shifts in isotherms (Sunday et al. 2015).

## Can a multidimensional perspective help disentangle pathways of community change?

Here, we illustrate how two important biodiversity responses to environmental change—community homogenization and local richness change (Dornelas et al. 2014, McGill et al. 2015)—are driven by fundamentally different pathways, rarely disentangled in the literature. We further discuss the potential for disaggregation of communities through time—a community response to climate change that can only be detected by studying the effects of temporal rather than spatial dimensions of temperature change.

### Disentangling community homogenization

Communities are generally becoming more similar in composition in time or space—a process called *homogenization*—often quantified as reduced beta diversity (Jurasinski and Kreyling 2007, Baiser et al. 2012, Davey et al. 2012, Savage and Vellend 2014, Magurran et al. 2015, but see Avolio et al. 2015). Identifying the specific dimension of temperature change leading to community homogenization has potential to help estimate the distinct effects of community abundance shifts (selection processes) versus species range shifts or expansions (movement processes, figure 4).

Selection processes cause community homogenization through time when a subset of species in a local assemblage systematically increase or decrease in abundance. For example, across many local stream-fish assemblages in France, temporal changes in community composition were related to losses of individuals—and, therefore, population declines and relative abundance changes—which has favored an increasingly similar set of species since the 1980s (Kuczynski et al. 2017). In this case, community homogenization was linked to selection processes and the timing dimension of temperature change emerged as an important predictor.

Through space, homogenization will occur when *the same* subset of species increasingly occupy many local communities across a region. For this to occur, species distributions must expand or contract by movement within a region. If range-shifting species display coordinated expansions or contractions across communities spatial homogenization is expected. This form of homogenization is often driven by movement of generalist species undergoing a range expansion (Davey et al. 2012). In cases in which movement processes drive homogenization, species richness will also increase (La Sorte 2006, Davey et al. 2012, Batt et al. 2017). However, the role of temperature position driving homogenization and richness is often unexplored (Davey et al. 2012, Savage and Vellend 2014). A pressing debate is the simultaneous stability of richness with ongoing biotic homogenization of communities (i.e., Magurran et al. 2015*cf.* Savage and Vellend 2014) and reordering of communities (e.g., Jones et al. 2017). This debate will benefit if the multiple dimensions of temperature change are identified in studies testing theory.

### Drivers of local richness change

Local species richness change is commonly attributed to the magnitude dimension of temperature change (Menéndez et al. 2006, Britton et al. 2009, Davey et al. 2012, Tayleur et al. 2015). However, increases in richness must occur because of species movements (i.e., local colonizations), whereas decreases can be due to selection (i.e., decline in situ) or movement (i.e., movement away from site). The rate that species move into a warmer (increased temperature magnitude) environment is determined in part by the position dimension of temperature change, but this could trade-off with greater species losses as warmer temperatures exceed species ­tolerance limits. Few studies, if any, have acknowledged the interaction between these two processes in driving species richness change.

### Community temporal disaggregation from changes in temporal position and availability of temperature

**Figure 6.**
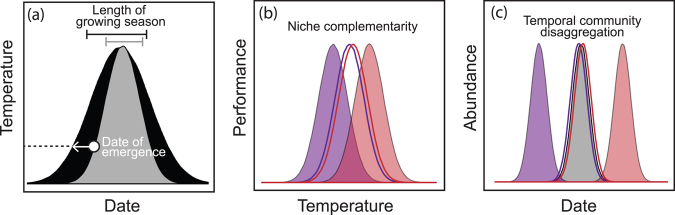
This schematic suggests how a community's response to temperature timing depends on the extent of overlap between thermal niches. (a) Temperature thresholds (the dashed line) for key demographic and physiological processes result in defined dates of first emergence and lengths of growing season, from time 1 (grey) to time 2 (black); changing the timing of temperature dimensions causes earlier emergence dates and an increased length of growing season. (b) Species niches in a community overlap to different extents; for example, niche complementarity is low in filled species thermal performance curves or niche complementarity is high in unfilled species thermal performance curves. (c) These differences result in community disaggregation between the timing of peak abundance for warm and cool species, as a result of the timing changes shown in panel (a).

Assessing community level responses to changes in timing dimension of temperature has revealed a distinct fingerprint on community composition, independent of changes in mean temperature. Specifically, Thomsen and colleagues (2015) found that with changing temporal position and availability of temperature, growing seasons are longer causing earlier springs and longer summer periods (these two dimensions were correlated in this study). These temperature changes affect the seasonal timing of peak abundances in the warm and cool affinity species differently. Warm species had later peak abundances, whereas cool species had earlier peak abundances (figure 6a). If communities have varying degrees of thermal niche complementarity—that is, high variation in thermal performance optima (figure 6b)—the temporal synchrony of species may breakdown. The extent of asynchrony will depend on species thermal trait distributions across the community (figure 6c). Long-term and high-resolution community time series are necessary to estimate the effects of temporal dimensions of temperature change, which may explain our gaps in understanding of community responses to this temperature dimension (Magurran et al. 2010, Thomsen et al. 2015).

## Conclusions

Similar ecological patterns can arise from different combinations of processes (Lawton 1999). We suggest this is also true when measuring community responses to environmental change. To understand biodiversity change on a warming Earth, we must link spatial and temporal structure of temperature and community change—short of this, misattribution of the climatic processes responsible for biodiversity change may occur. Similar community changes can occur through both selection and movement pathways; biodiversity forecasts and management decisions may depend on the relative importance of each. Characterizing the dimensionality of how temperature is changing at scales relevant for biodiversity processes will require closer collaboration between physical scientists and ecologists. This will hopefully lead to an attribution of temperature's effects beyond average temperature change. We demonstrate a need to build these mechanistic connections into how physical regimes affect biodiversity change, being explicit in space and time.

## Supplementary Material

Supplemental material

## References

[bib1] Alexander JM, Diez JM, Levine JM 2015 Novel competitors shape species’ responses to climate change. Nature 525: 515–518. 26374998 10.1038/nature14952

[bib2] Angilletta MJ. 2009 Thermal Adaptation: A Theoretical and Empirical Synthesis. Oxford University Press, Oxford.

[bib3] Avolio ML, La Pierre KJ, Houseman GR 2015 A framework for quantifying the magnitude and variability of community responses to global change drivers. Ecosphere 6: 1–14.

[bib4] Baiser B, Olden JD, Record S 2012 Pattern and process of biotic homogenization in the New Pangaea. Proceedings of the Royal Society B 279: 4772–4777. 23055062 10.1098/rspb.2012.1651 PMC3497087

[bib5] Barnosky AD. 2008 Megafauna biomass tradeoff as a driver of Quaternary and future extinctions. Proceedings of the National Academy of Sciences 105: 11543–11548. 10.1073/pnas.0801918105 PMC2556404 18695222

[bib6] Bates AE, Barrett NS, Stuart-Smith RD 2014a Resilience and signatures of tropicalization in protected reef fish communities. Nature Climate Change 4: 62–67.

[bib7] Bates AE, Pecl GT, Frusher S 2014b Defining and observing stages of climate-mediated range shifts in marine systems. Global Environmental Change 26: 27–38.

[bib8] Batt RD, Morley JW, Selden RL, Morgan W 2017 Gradual changes in range size accompany long-term trends in species richness. Ecology Letters 20: 1148–1157. doi:10.1111/ele.12812. 28699209 10.1111/ele.12812

[bib9] Bellard C, Bertelsmeier C, Leadley P 2012 Impacts of climate change on the future of biodiversity. Ecology Letters 15: 365–377. 22257223 10.1111/j.1461-0248.2011.01736.x PMC3880584

[bib10] Borregaard MK, Rahbek C 2010 Causality of the relationship between geographic distribution and species abundance. The Quarterly review of biology 85: 253–291. 20337258 10.1086/650265

[bib11] Both C, Van Asch M, Bijlsma RG 2009 Climate change and unequal phenological changes across four trophic levels: Constraints or adaptations? Journal of Animal Ecology 78: 73–83. 18771506 10.1111/j.1365-2656.2008.01458.x

[bib12] Britton AJ, Beale CM, Towers W, Hewison RL 2009 Biodiversity gains and losses: Evidence for homogenisation of Scottish alpine vegetation. Biological conservation 142: 1728–1739.

[bib13] Brose U, Ostling A, Harrison K, Martinez ND 2004 Unified spatial scaling of species and their trophic interactions. Nature 428: 167–171. 15014497 10.1038/nature02297

[bib14] Burrows M, Schoeman DS, Buckley LB 2011 The pace of shifting climate in marine and terrestrial ecosystems. Science 334: 652–655. 22053045 10.1126/science.1210288

[bib15] Cline TJ, Bennington V, Kitchell JF 2013 Climate Change Expands the Spatial Extent and Duration of Preferred Thermal Habitat for Lake Superior Fishes. PLoS ONE 8: e62279. 23638023 10.1371/journal.pone.0062279 PMC3637403

[bib16] Davey CM, Chamberlain DE, Newson SE 2012 Rise of the generalists: Evidence for climate driven homogenization in avian communities. Global Ecology and Biogeography 21: 568–578.

[bib17] Dell AI, Pawar S, Savage VM 2011 Systematic variation in the temperature dependence of physiological and ecological traits. Proceedings of the National Academy of Sciences 108: 10591–10596. 10.1073/pnas.1015178108 PMC3127911 21606358

[bib18] Devictor V, Julliard R, Couvet D, Jiguet F 2008 Birds are tracking climate warming, but not fast enough. Proceedings of the Royal Society B 275: 2743–2748. 18713715 10.1098/rspb.2008.0878 PMC2605823

[bib19] Devictor V, van Swaay C, Brereton T 2012 Differences in the climatic debts of birds and butterflies at a continental scale. Nature Climate Change 2: 121–124.

[bib20] Dornelas M, Gotelli NJ, McGill B 2014 Assemblage time series reveal biodiversity change but not systematic loss. Science (New York, NY) 344: 296–9. 10.1126/science.1248484 24744374

[bib21] Dowd WW, King FA, Denny MW 2015 Thermal variation, thermal extremes and the physiological performance of individuals. Journal of Experimental Biology 218: 1956–1967. 26085672 10.1242/jeb.114926

[bib22] Durner G, Douglas D, Nielson R 2009 Predicting 21st-century polar bear habitat distribution from global climate models. Ecological Monographs 79: 25–58.

[bib23] Fauth JE, Bernado J, Camara M 1996 Simplifying the jargon of community ecology: A conceptual approach. The American Naturalist 147: 282–286.

[bib24] Gaillard JM, Mark Hewison AJ, Klein F 2013 How does climate change influence demographic processes of widespread species? Lessons from the comparative analysis of contrasted populations of roe deer. Ecology Letters 16: 48–57. 23297773 10.1111/ele.12059

[bib25] Garcia RA, Cabeza M, Rahbek C, Araujo MB 2014 Multiple dimensions of climate change and their implications for biodiversity. Science 344: 1247579–1247579. 24786084 10.1126/science.1247579

[bib26] Gaüzère P, Jiguet F, Devictor V 2015 Rapid adjustment of bird community compositions to local climatic variations and its functional consequences. Global Change Biology 21: 3367–3378. 25731935 10.1111/gcb.12917

[bib27] Gilman SE, Urban MC, Tewksbury J 2010 A framework for community interactions under climate change. Trends in Ecology and Evolution 25: 325–331. 20392517 10.1016/j.tree.2010.03.002

[bib28] Hamann A, Roberts DR, Barber QE 2015 Velocity of climate change algorithms for guiding conservation and management. Global Change Biology 21: 997–1004. 25310933 10.1111/gcb.12736

[bib29] Hanski I. 1998 Metapopulation dynamics. Nature 396: 41–49.

[bib30] Hawkins B, Field R, Cornell H 2003 Energy, water, and broad-scale geographic patterns of species richness. Ecology 84: 3105–3117.

[bib31] Hiddink JG, Burrows MT, García Molinos J 2015 Temperature tracking by North Sea benthic invertebrates in response to climate change. Global Change Biology 21: 117–129. 25179407 10.1111/gcb.12726

[bib32] Hill SLL, Harfoot M, Purvis A 2016 Reconciling Biodiversity Indicators to Guide Understanding and Action. Conservation Letters 9: 405–412.

[bib33] Hofreiter M, Stewart J 2009 Ecological change, range fluctuations and population dynamics during the pleistocene. Current Biology 19: R584–R594. 19640497 10.1016/j.cub.2009.06.030

[bib34] Horta E, Costa B, Assis J, Franco G 2014 Tropicalization of fish assemblages in temperate biogeographic transition zones. Marine Ecology Progress Series 504: 241–252.

[bib35] Houlahan JE, Mckinney ST, Anderson TM, Mcgill BJ 2017 The priority of prediction in ecological understanding. Oikos 126: 1–7. doi:10.1111/ oik.03726

[bib36] [IPCC] Intergovernmental Panel on Climate Change 2013 Climate Change 2013: The Physical Science Basis. Summary for Policymakers. IPCC.

[bib37] Johnston A, Ausden M, Dodd AM 2013 Observed and predicted effects of climate change on species abundance in protected areas. Nature Climate Change 3: 1055–1061.

[bib38] Jones VP, Wiman NG 2012 Modeling the interaction of physiological time, seasonal weather patterns, and delayed mating on population dynamics of codling moth, Cydia pomonella (L.; Lepidoptera: Tortricidae). Population Ecology 54: 421–429.

[bib39] Jones SK, Ripplinger J, Collins SL 2017 Species reordering, not changes in richness, drives long-term dynamics in grassland communities. Ecology Letters 20: 1556–1565. 29027343 10.1111/ele.12864

[bib40] Jurasinski G, Kreyling J 2007 Upward shift of alpine plants increases floristic similarity of mountain summarymits. Journal of Vegetation Science 18: 711–718.

[bib41] Kock RA, Orynbayev M, Robinson S 2018 Saigas on the brink: Multidisciplinary analysis of the factors influencing mass mortality events. Science Advances 4: eaao2314. 29376120 10.1126/sciadv.aao2314 PMC5777396

[bib42] Kordas RL, Harley CDG, O’Connor MI 2011 Community ecology in a warming world: The influence of temperature on interspecific interactions in marine systems. Journal of Experimental Marine Biology and Ecology 400: 218–226.

[bib43] Kuczynski L, Legendre P, Grenouillet G 2017 Concomitant impacts of climate change, fragmentation and non-native species have led to reorganization of fish communities since the 1980s. Global Ecology and Biogeography 27: 1–10. doi:10.1111/geb.12690.

[bib44] La Sorte FA. 2006 Geographical expansion and increase prevalence of commom species in avian assemblages: Implications for large-scale patterns of species richness. Journal of Biogeography 33: 1183–1191.

[bib45] Lawton J. 1999 Are there general laws in ecology? Oikos 84: 177–192.

[bib46] Lenoir J, Svenning JC 2015 Climate-related range shifts - a global multidimensional synthesis and new research directions. Ecography 38: 15–28.

[bib47] Lichter J. 2000 Colonization constraints during primary succession on coastal Lake Michigan sand dunes. Journal of Ecology 88: 825–839.

[bib48] Loarie SR, Duffy PB, Hamilton H 2009 The velocity of climate change. Nature 462: 1052–1055. 20033047 10.1038/nature08649

[bib49] Lyons SK, Smith FA, Brown JH 2004 Of mice, matodons and men: Human-mediated extinctions on four cotinents. Evolutionary Ecology Research 6: 339–358.

[bib50] MacArthur R, Wilson EO 1967 The Theory of Island Biogeography. Princeton University Press.

[bib51] Magnuson JJ, Crowder LB, Medvick PA 1979 Temperature as an ecological resource. American Zoologist 19: 331–343.

[bib52] Magurran AE, Baillie SR, Buckland ST 2010 Long-term datasets in biodiversity research and monitoring: Assessing change in ecological communities through time. Trends in Ecology and Evolution 25: 574–582. 20656371 10.1016/j.tree.2010.06.016

[bib53] Magurran AE, Dornelas M, Moyes F 2015 Rapid biotic homogenization of marine fish assemblages. Nature Communications 6: 8405. 10.1038/ncomms9405 PMC4598618 26400102

[bib54] Magurran AE, McGill B 2011 Biological Diversity: Frontiers in Measurement and Assessment. Oxford University Press, Oxford.

[bib55] Makoto K, Wilson SD 2016 New Multicentury Evidence for Dispersal Limitation during Primary Succession. The American Naturalist 187: 804–811. 10.1086/686199 27172599

[bib56] Matechou E, Dennis EB, Freeman SN, Brereton T 2014 Monitoring abundance and phenology in (multivoltine) butterfly species: A novel mixture model. Journal of Applied Ecology 51: 766–775.

[bib57] Matthiopoulos J, Fieberg J, Aarts G 2015 Establishing the link between habitat-selection and animal population dynamics. Ecological Monographs 85: 150119094816002.

[bib58] McGill BJ, Dornelas M, Gotelli NJ, Magurran AE 2015 Fifteen forms of biodiversity trend in the Anthropocene. Trends in Ecology and Evolution 30: 104–113. 25542312 10.1016/j.tree.2014.11.006

[bib59] Mckechnie AE, Wolf BO 2010 Climate change increases the likelihood of catastrophic avian mortality events during extreme heat waves. Biology Letters 6: 253–256. 19793742 10.1098/rsbl.2009.0702 PMC2865035

[bib60] McMahon CR, Hays GC 2006 Thermal niche, large-scale movements and implications of climate change for a critically endangered marine vertebrate. Global Change Biology 12: 1330–1338.

[bib61] Melville J. 2002 Competition and character displacement in two species of scincid lizards. Ecology Letters 5: 386–393.

[bib62] Menéndez R, Megías AG, Hill JK 2006 Species richness changes lag behind climate change. Proceedings of the Royal Society B 273: 1465–1470. 16777739 10.1098/rspb.2006.3484 PMC1560312

[bib63] Nogues-Bravo D, Ohlemuller R, Batra P, Araujo MB 2010 Climate predictors of late quaternary extinctions. Evolution 64: 2442–2449. 20707809 10.1111/j.1558-5646.2010.01009.x

[bib64] O’Connor MI, Holding JM, Kappel CV 2015 Strengthening confidence in climate change impact science. Global Ecology and Biogeography 24: 64–76.

[bib65] Ordonez A, Williams JW 2013 Projecting climate reshuffling based on multivariate cliamte-availability, climate-analog, and climate-velocity analyses: Implications for climate disaggregation. Climate Change 119: 659–675.

[bib66] Payne N, Meyer CG, Smith JA 2018 Combining abundance and performance data reveals how temperature regulates coastal occurrences and activity of a roaming apex predator. Global Change Biology 24: 1884–1893. doi: 10.1111/gcb.14088 29516588 10.1111/gcb.14088

[bib67] Payne NL, Smith JA, van der Meulen DE 2016 Temperature dependence of fish performance in the wild: Links with species biogeography and physiological thermal tolerance. Functional Ecology 30: 903–912.

[bib68] Pinsky ML, Worm B, Fogarty MJ 2013 Marine taxa track local climate velocities. Science 341: 1239–1242. 24031017 10.1126/science.1239352

[bib69] Purvis A, Gittleman JL, Cowlishaw G, Mace GM 2000 Predicting extinction risk in declining species. Proceedings of the Royal Society B 267: 1947–1952. 11075706 10.1098/rspb.2000.1234 PMC1690772

[bib70] Radeloff VC, Williams JW, Bateman BL 2015 The rise of novelty in ecosystems. Ecological Applications 25: 2051–2068. doi:10.1890/14-1781.1 26910939 10.1890/14-1781.1

[bib71] Roughgarden J, Porter W, Heckel D 1981 Resource partitioning of space and its relationship to body temperature in Anolis lizard populations. Oecologia 50: 256–264. 28311098 10.1007/BF00348048

[bib72] Runjie Z, Heong KL, Domingo IT 1996 Relationship between temperature and functional response in Cardiochiles philippinensis (Hymenoptera: Braconidae), a larvae parasitoid of Cnaphalocrocis medinalis (Lepidoptera: Pyralidae). Environmental Entomology 25: 1321–1324.

[bib73] Santini L 2016 Assessing the suitability of diversity metrics to detect biodiversity change. Biological Conservation 213: 341–350.

[bib74] Savage J, Vellend M 2014 Elevational shifts, biotic homogenization and time lags in vegetation change during 40 years of climate warming. Ecography 37: 001–010.

[bib75] Sax DF, Gaines SD 2008 Species invasions and extinction: The future of native biodiversity on islands. Proceedings of the National Academy of Sciences 105: 11490–11497. 10.1073/pnas.0802290105 PMC2556416 18695231

[bib76] Senior RA, Hill JK, González P, Edwards DP 2017 A pantropical analysis of the impacts of forest degradation and conversion on local temperature. Ecological Evolution 7: 7897–7908. 10.1002/ece3.3262 PMC5632667 29043043

[bib77] Smith MD, Knapp AK, Collins SL 2009 A framework for assessing ecosystem dynamics in response to chronic resource alterations induced by global change. Ecology 90: 3279–3289. 20120798 10.1890/08-1815.1

[bib78] Stephens PA, Mason LR, Green RE 2016 Consistent response of bird populations to climate change on two continents. Science 352: 84–87. 27034371 10.1126/science.aac4858

[bib79] Sunday JM, Pecl GT, Frusher S 2015 Species traits and climate velocity explain geographic range shifts in an ocean-warming hotspot. Ecology Letters 18: 944–953. 26189556 10.1111/ele.12474

[bib80] Tayleur CM, Devictor V, Gaüzère P 2015 Regional variation in climate change winners and losers highlights the rapid loss of cold-dwelling species. Diversity and Distributions 22: 1–13.

[bib81] ter Hofstede R, Hiddink J, Rijnsdorp A 2010 Regional warming changes fish species richness in the eastern North Atlantic Ocean. Marine Ecology Progress Series 414: 1–9.

[bib82] Thackeray SJ, Sparks TH, Frederiksen M 2010 Trophic level asynchrony in rates of phenological change for marine, freshwater and terrestrial environments. Global Change Biology 16: 3304–3313.

[bib83] Thomsen PF, Jorgensen PS, Bruun HH 2015 Resource specialists lead local insect community turnover associated with temperature - analysis of an 18-year full-seasonal record of moths and beetles. Journal of Animal Ecology 85: 251–261. 26521706 10.1111/1365-2656.12452

[bib84] Tuff KT, Tuff T, Davies KF 2016 A framework for integrating thermal biology into fragmentation research. Ecology Letters 19: 361–374. 26892491 10.1111/ele.12579 PMC4794773

[bib85] Urban MC, Tewksbury JJ, Sheldon KS 2012 On a collision course: Competition and dispersal differences create no-analogue communities and cause extinctions during climate change. Proceedings of the Royal Society B 279: 2072–2080. 22217718 10.1098/rspb.2011.2367 PMC3311897

[bib86] Vellend M. 2010 Conceptual synthesis in community ecology. The Quarterly Review of Biology 85: 183–206. 20565040 10.1086/652373

[bib87] Vellend M. 2016 The Theory of Ecological Communities, 1st ed Princeton University Press.

[bib88] Vellend M, Baeten L, Becker-Scarpitta A 2017 Plant Biodiversity Change Across Scales During the Anthropocene. Annual Review of Plant Biology 68: 563–586. 10.1146/annurev-arplant-042916-040949 28125286

[bib89] Wang G, Dillon ME 2014 Recent geographic convergence in diurnal and annual temperature cycling flattens global thermal profiles. Nature Climate Change 4: 988–992.

[bib90] Williams JW, Jackson ST, Kutzbach JE 2007 Projected distributions of novel and disappearing climates by 2100 AD. Proceedings of the National Academy of Sciences 104: 5738–5742. 10.1073/pnas.0606292104 PMC1851561 17389402

