# Supplementary material for: Temperature-Driven Biodiversity Change: Disentangling Space and Time
Source: Bioscience. 2018 Sep 19;68(11):873–84. doi: 10.1093/biosci/biy096 (PMC6238962; doi:10.1093/biosci/biy096)
Supplement: Supplemental material [file biy096_supplemental_files.docx]

**Supplemental Materials: A summary of literature review methods, results, discussion, Figure S1 and references.**

**Literature review: Method**

A literature review was undertaken by searching ‘Web of Knowledge’ for studies published between 2005 and 2015, for multiple taxonomic groups (bird, mammal, plant, fish, invertebrates, insect, amphibian, reptile, marine mammal) the topic was refined to combinations of (‘climate’ OR ‘temperature’) AND (‘change’ OR ‘increase’ ‘variation’ OR ‘anomaly’ OR ‘extreme’ OR ‘seasonality’) with the additional search field of (‘diversity’ OR ‘biodiversity’) AND (‘response’ OR ‘change’ OR ‘loss’). A second search was undertaken using (‘climate’ NEAR (‘change’ OR ‘variability’ OR ‘velocity’)) NEAR ((alpha OR beta OR gamma) NEAR diversity). The popular search teams such as ‘climate change’ and ‘biodiversity’ resulted in a wealth of literature (n=2735) therefore, whilst community wasn’t specifically searched for (due to multiple definitions across different fields for ‘community’) the broader and more popular keyword ‘biodiversity’ encompassed most relevant ecological studies of communities. Additional relevant literature was identified from the reference lists from the retrieved studies. From this literature search articles were removed if they did not fit the following criteria: (i) linking change in temperature parameters to community response (not population, individual or species level), (ii) observational studies should be recent influence of temperature change (<150 years) rather than over geological time frames, (iii) temperature parameter should be considered as driving a temporal change in communities. This resulted in 156 peer-reviewed articles from which study attributes were determined to understand current coverage of temperature dimensions in the literature. We recorded the dimensions of temperature used in analyses (based on our glossary definitions in the main manuscript), taxonomic scope, research method (observation, experiment or model), and community response measured to assess research coverage over the past decade.

**Literature review: Summary**

We identified 156 relevant peer-reviewed articles, from a total of 2735 articles, that were returned from Web of Knowledge searches. To reiterate from the main paper, from the 156 papers returned from our literature review we find that 86% focus on temperature magnitude, and a tiny proportion of studies investigate spatial position (3%) and availability (1%) or temporal position (4%) and availability (6%, figure 2 main manuscript). Of the metrics used to measure magnitude - the statistical distribution of temperature change - mean changes were investigated in 48% studies, and 41% of studies focussed on minimum or maximum temperatures (figure S1a). These biases show little sign of lessening through time (figure S1b).

We also find significant information gaps. Most of the returned literature focussed on a few well-studied taxonomic groups (plants, 39%; marine fishes, 20%; birds, 17%). Some charismatic groups (mammals, 1%) and functionally important taxa are highly under-represented (e.g., phytoplankton, <1%; insects, 7%). In addition to taxonomic gaps, there is limited application of different community metrics to quantify community responses to temperature change. Generally, metrics such as species richness (36%), species identity (13%) and species relative abundance (6%) are frequently used, while phylogenetic metrics (<1%) remain poorly examined (supplemental materials figure S1c). Diversity metrics, which are known to provide insensitive estimates of temporal community changes (Magurran and Henderson 2010), are commonly selected as a response metric (17%).

**Literature review: Discussion**

1. *Magnitude: Mean temperature*

In reviewing the literature, we find that changes in mean temperature predict changes in composition of fish communities (Bates et al. 2014, Magurran et al. 2015), as well as birds, plants, and invertebrates (Roth et al. 2014). Compositional responses to mean temperature are as expected when selection alters species relative abundance in a deterministic manner. A commonly documented response to warming was an increase in communities’ average temperature-affinity (Community Temperature Index, CTI), across broad variety of taxa and habitats (e.g. Devictor et al. 2012, Bates et al. 2014, Duque et al. 2015, Gaüzère et al. 2015, Tayleur et al. 2015). The link between temperature and community warming, measured using the realized niche, supports the expected role of selection community processes filtering for warm-adapted species, which in turn drives trends in community composition in response to average temperature conditions.

In contrast to composition and trait-based community changes, evidence for species richness being tightly linked to mean temperatures is weaker. For example, the direction of change in richness with warming is variable, with frequent documentation of no effects (Menéndez et al. 2006, Magurran et al. 2015, Tayleur et al. 2015) and positive effects (Hiddink and ter Hofstede 2008, Davey et al. 2012).

1. *Magnitude: Extreme events*

Extreme events are defined by statistically extreme and temporally abrupt periods of temperature, relative to the temperature variability experienced over an organism’s life-cycle. These discrete events often have disproportional effects on organisms relative to the duration of the event and the effects of an underlying long-term trend in temperature (Jentsch et al. 2007, Smith 2011, Bailey and van de Pol 2015). Variation in the inherent sensitivity of communities, in combination with the short and infrequent nature of such events, means that predicting and measuring the impact of extreme events can be challenging (Smith 2011). At a population level, temperature extremes have been generally shown to cause drastic abundance declines of species’ populations (between 13% to 19% decline in bird abundances, Albright et al. 2010; 100% cover reduction in macroalgae, Smale and Wernberg 2013. The effects are worsened in regionally extensive extreme events, such as the European 2003 heatwave, where movement cannot facilitate avoidance of physiological damaging temperatures. In these events, mortality and demographic changes are extreme (Jiguet et al. 2006, Garrabou et al. 2009), particularly for sessile species (Mouthon and Daufresne 2006).

Community relative abundance responses to extreme events are much more responsive than richness changes in bird species across the USA (Albright et al. 2011). In contrast, large scale richness losses were found for freshwater molluscs when abundance declines are high enough to causing local extinctions, because of limited movement capability relative to the duration of the extreme event in this taxa (Mouthon and Daufresne 2006). Species’ abundance changes are generally directional with respect to thermal affinity (Day et al., Jiguet et al. 2006, Boucek and Rehage 2014, Wernberg et al. 2016), and can have drastic impacts on ecosystem structure and services beyond the community scale effects ((Pratchett et al. 2011, Butt et al. 2015, Wernberg et al. 2016)). However, community responses vary greatly between studies, which is expected because community responses to extreme events depend on: i) how extreme events alter species interactions (Butt et al. 2015), ii) historical contingencies in community composition prior to extreme events (i.e., priority effects), iii) and post-disturbance chance colonisations post-extreme events (i.e., ecological legacy effects, Seifert et al. 2015).

1. *Temporal availability and position*

At a species level, responses to the temporal dimensions of temperature change are frequently documented but our review demonstrates that evidence is limited for community-scale responses. Meta-analyses of species-level responses to temperature timing suggest widespread earlier timing of life-history events, migration, and date of emergence in marine (4.4 days per decade, Poloczanska et al. 2013) and terrestrial (2.3-2.8 days per decade, Parmesan and Yohe 2003) systems. We found only one observational study of whole community responses to temporal position of temperature change, Thomsen et al. 2015). In this study, advancing species abundance is linked to species thermal tolerance limits, highlighting a role for selection processes driving community compositional responses. This effect occurs because timing affects fitness peaks throughout the year which depends on species thermal preferences.

1. *Availability*

Our review suggests that when quantifying community responses to changes in temperature availability across space, even a basic foundation is lacking Our review suggests few, if any, observational studies exist that document regional or local community responses to recent changes in temperature availability (i.e., novel climates, changing size and shape of preferred temperatures). This is an important knowledge gap given that from 4% to 20% of the Earth’s terrestrial surface is expected to be affected by changing temperature availability by 2100 (specifically novel climates, Williams and Jackson 2007). Multi-species distribution models and theoretical explorations have, however, identified a role of emerging novel temperature regimes in driving community turnover through new species groups assembling in this novel temperature niche space (Stralberg et al. 2009).

1. *Position*

Indicative of species’ movements being an essential process driving richness gains, projections from species distribution shifts in birds suggest species richness gains will occur across the United States of America (Bateman et al. 2016). In contrast, historic high rates of forward velocity after the last glacial maximum (i.e., the distance to suitable sites post-warming) were not perfectly tracked by species movements. This inability to track change in the spatial position of temperature likely explains local extinctions, particularly for species with small ranges and endemics, in climatically unstable regions, contributing to latitudinal variation in species richness (Sandel et al. 2011). Data from contemporary biological communities suggest species movements may lag behind the rate of temperature velocity (perhaps due to spatial variation in temperature leading to climate refugia and slowing responses to the position dimension of temperature change). For example, across a range of terrestrial taxa, the average thermal affinity of species in a community (CTI) increases directionally with temperature velocity, but at a slower rate than necessary to maintain constant temperatures (Devictor et al. 2008, 2012).

Figure S1. Summary plots from literature review. ((b) Aspects of magnitude dimension studied in explaining community responses. (c) Metrics commonly used to study observational responses to temperature change, richness based metrics are most common. Number of articles published for each dimension and year across all dimensions.

**Literature review references:**

| 1. Alatalo JM, Little CJ, Jägerbrand AK, Molau U. 2014. Dominance hierarchies, diversity and species richness of vascular plants in an alpine meadow: contrasting short and medium term responses to simulated global change. PeerJ. doi: 10.7717/peerj.406 |
| --- |
| 1. Albouy C, Guilhaumon F, Araujo MB, et al. 2012. Combining projected changes in species richness and composition reveals climate change impacts on coastal Mediterranean fish assemblages. Global Change Biology 18:2995–3003. doi: 10.1111/j.1365-2486.2012.02772.x |
| 1. Albouy C, Guilhaumon F, Leprieur F, et al. 2013. Projected climate change and the changing biogeography of coastal Mediterranean fishes. Journal of Biogeography 40:534–547. doi: 10.1111/jbi.12013 |
| 1. Albouy C, Leprieur F, Le Loc’h F, et al. 2015. Projected impacts of climate warming on the functional and phylogenetic components of coastal Mediterranean fish biodiversity. Ecography 38:681–689. doi: 10.1111/ecog.01254 |
| 1. Alvarez-Fernandez S, Lindeboom H, Meesters E. 2012. Temporal changes in plankton of the North Sea: community shifts and environmental drivers. Marine Ecology Progress Series 462:21–38. doi: 10.3354/meps09817 |
| 1. Aschan M, Fossheim M, Greenacre M, Primicerio R. 2013. Change in Fish Community Structure in the Barents Sea. PLoS ONE 8:e62748. doi: 10.1371/journal.pone.0062748 |
| 1. Auber A, Travers-Trolet M, Villanueva MC, Ernande B. 2015. Regime Shift in an Exploited Fish Community Related to Natural Climate Oscillations. PLoS ONE 10:e0129883. doi: 10.1371/journal.pone.0129883 |
| 1. Bassler C, Hothorn T, Brandl R, Mueller J. 2013. Insects Overshoot the Expected Upslope Shift Caused by Climate Warming. PLoS ONE 8:e65842. doi: 10.1371/journal.pone.0065842 |
| 1. Bakkenes M, Eickhout B, Alkemade R. 2006. Impacts of different climate stabilisation scenarios on plant species in Europe. Global Environmental Change-Human and Policy Dimensions 16:19–28. doi: 10.1016/j.gloenvcha.2005.11.001 |
| 1. Baldwin AH, Jensen K, Schoenfeldt M. 2014. Warming increases plant biomass and reduces diversity across continents, latitudes, and species migration scenarios in experimental wetland communities. Global Change Biology 20:835–850. |
| 1. Barbet-Massin M, Thuiller W, Jiguet F. 2012. The fate of European breeding birds under climate, land-use and dispersal scenarios. Global Change Biology 18:881–890. doi: 10.1111/j.1365-2486.2011.02552.x |
| 1. Bates AE, Barrett NS, Stuart-Smith RD, et al. 2014. Resilience and signatures of tropicalization in protected reef fish communities. Nature Climate Change 4:62–67. doi: 10.1038/nclimate2062 |
| 1. Beaugrand G, Luczak C, Edwards M. 2009. Rapid biogeographical plankton shifts in the North Atlantic Ocean. Global Change Biology 15:1790–1803. doi: 10.1111/j.1365-2486.2009.01848.x |
| 1. Bernhardt-Roemermann M, Baeten L, Craven D, et al. 2015. Drivers of temporal changes in temperate forest plant diversity vary across spatial scales. Global Change Biology 21:3726–3737. doi: 10.1111/gcb.12993 |
| 1. Blanchard JL, Dulvy NK, Jennings S, et al. 2005. Do climate and fishing influence size-based indicators of Celtic Sea fish community structure? Ices Journal of Marine Science 62:405–411. doi: 10.1016/j.icesjms.2005.01.006 |
| 1. Bokhorst S, Huiskes A, Convey P, Aerts R. 2007. The effect of environmental change on vascular plant and cryptogam communities from the Falkland Islands and the Maritime Antarctic. BMC Ecol. doi: 10.1186/1472-6785-7-15 |
| 1. Boulangeat I, Georges D, Dentant C, et al. 2014. Anticipating the spatio-temporal response of plant diversity and vegetation structure to climate and land use change in a protected area. Ecography 37:1230–1239. doi: 10.1111/ecog.00694 |
| 1. Brodeur RD, Peterson WT, Auth TD, et al. 2008. Abundance and diversity of coastal fish larvae as indicators of recent changes in ocean and climate conditions in the Oregon upwelling zone. Marine Ecology Progress Series 366:187–202. doi: 10.3354/meps07539 |
| 1. Brooker RW, Matesanz S, Valladares F, Klotz S. 2012. Long-term spatial pattern change in a semi-arid plant community: The role of climate and composition. Acta Oecologica-International Journal of Ecology 45:8–15. doi: 10.1016/j.actao.2012.08.002 |
| 1. Brown KA, Parks KE, Bethell CA, et al. 2015. Predicting Plant Diversity Patterns in Madagascar: Understanding the Effects of Climate and Land Cover Change in a Biodiversity Hotspot. PLoS ONE 10:e0122721. doi: 10.1371/journal.pone.0122721 |
| 1. Buchheister A, Bonzek CF, Gartland J, Latour RJ. 2013. Patterns and drivers of the demersal fish community of Chesapeake Bay. Marine Ecology Progress Series 481:161–180. doi: 10.3354/meps10253 |
| 1. Buisson L, Thuiller W, Lek S, et al. 2008. Climate change hastens the turnover of stream fish assemblages. Global Change Biology 14:2232–2248. doi: 10.1111/j.1365-2486.2008.01657.x |
| 1. Buisson L, Grenouillet G. 2009. Contrasted impacts of climate change on stream fish assemblages along an environmental gradient. Diversity and Distributions 15:613–626. doi: 10.1111/j.1472-4642.2009.00565.x |
| 1. Buisson L, Grenouillet G, Villeger S, et al. 2013. Toward a loss of functional diversity in stream fish assemblages under climate change. Global Change Biology 19:387–400. doi: 10.1111/gcb.12056 |
| 1. Burrows JA, Harvey JT, Newton KM, et al. 2012. Marine mammal response to interannual variability in Monterey Bay, California. Marine Ecology Progress Series 461:257–271. doi: 10.3354/meps09712 |
| 1. Cartes JE, Maynou F, Fanelli E, et al. 2015. Changes in deep-sea fish and crustacean communities at 1000-2200 m in the Western Mediterranean after 25 years: Relation to hydro-climatic conditions. Journal of Marine Systems 143:138–153. doi: 10.1016/j.jmarsys.2014.10.015 |
| 1. Chollet S, Rambal S, Fayolle A, et al. 2014. Combined effects of climate, resource availability, and plant traits on biomass produced in a Mediterranean rangeland. Ecology 95:737–748. doi: 10.1890/13-0751.1 |
| 1. Chou CH, Huang TJ, Lee YP, et al. 2011. Diversity of the alpine vegetation in central Taiwan is affected by climate change based on a century of floristic inventories. Botanical Studies 52:503–516. |
| 1. Chu C, Jones NE, Mandrak NE, et al. 2008. The influence of air temperature, groundwater discharge, and climate change on the thermal diversity of stream fishes in southern Ontario watersheds. Canadian Journal of Fisheries and Aquatic Sciences 65:297–308. doi: 10.1139/f08-007 |
| 1. Chung S, Suzaki H, Kasai A, Nakata H. 2015. The Response of Fish Communities to Climate and Human-Induced Changes Inferred from Fishery Landings in an Enclosed Bay. Estuaries and Coasts 38:1365–1375. doi: 10.1007/s12237-014-9884-4 |
| 1. Coetzee BWT, Robertson MP, Erasmus BFN, et al. 2009. Ensemble models predict Important Bird Areas in southern Africa will become less effective for conserving endemic birds under climate change. Global Ecology and Biogeography 18:701–710. doi: 10.1111/j.1466-8238.2009.00485.x |
| 1. Collie JS, Wood AD, Jeffries HP. 2008. Long-term shifts in the species composition of a coastal fish community. Canadian Journal of Fisheries and Aquatic Sciences 65:1352–1365. doi: 10.1139/f08-048 |
| 1. Conti L, Comte L, Hugueny B, Grenouillet G. 2015. Drivers of freshwater fish colonisations and extirpations under climate change. Ecography 38:510–519. doi: 10.1111/ecog.00753 |
| 1. Danby RK, Koh S, Hik DS, Price LW. 2011. Four Decades of Plant Community Change in the Alpine Tundra of Southwest Yukon, Canada. Ambio 40:660–671. doi: 10.1007/s13280-011-0172-2 |
| 1. Daufresne M, Boet P. 2007. Climate change impacts on structure and diversity of fish communities in rivers. Global Change Biology 13:2467–2478. doi: 10.1111/j.1365-2486.2007.01449.x |
| 1. Daufresne M, Veslot J, Capra H, et al. 2015. Fish community dynamics (1985-2010) in multiple reaches of a large river subjected to flow restoration and other environmental changes. Freshwater Biology 60:1176–1191. doi: 10.1111/fwb.12546 |
| 1. Davey CM, Chamberlain DE, Newson SE, et al. 2012. Rise of the generalists: evidence for climate driven homogenization in avian communities. Global Ecology and Biogeography 21:568–578. doi: 10.1111/j.1466-8238.2011.00693.x |
| 1. Davey CM, Devictor V, Jonzén N, et al. 2013. Impact of climate change on communities: revealing species’ contribution. Journal of Animal Ecology 82:551–561. doi: 10.1111/1365-2656.12035 |
| 1. De Frenne P, Rodriguez-Sanchez F, Coomes DA, et al. 2013. Microclimate moderates plant responses to macroclimate warming. Proceedings of the National Academy of Sciences of the United States of America 110:18561–18565. doi: 10.1073/pnas.1311190110 |
| 1. Del Vecchio S, Prisco I, Acosta ATR, Stanisci A. 2015. Changes in plant species composition of coastal dune habitats over a 20-year period. AoB Plants 7:plv018. doi: 10.1093/aobpla/plv018 |
| 1. de Sassi C, Lewis OT, Tylianakis JM. 2012. Plant-mediated and nonadditive effects of two global change drivers on an insect herbivore community. Ecology 93:1892–1901. |
| 1. de Souza TV, Lorini ML, Alves MAS, et al. 2011. Redistribution of Threatened and Endemic Atlantic Forest Birds Under Climate Change. Natureza & Conservacao 9:214–218. doi: 10.4322/natcon.2011.028 |
| 1. Devictor V, Julliard R, Couvet D, Jiguet F. 2008. Birds are tracking climate warming, but not fast enough. Proceedings Biological sciences / The Royal Society 275:2743–2748. doi: 10.1098/rspb.2008.0878 |
| 1. Devictor V, van Swaay C, Brereton T, et al. 2012. Differences in the climatic debts of birds and butterflies at a continental scale. Nature Climate Change 2:121–124. doi: 10.1038/nclimate1347 |
| 1. Dieleman CM, Branfireun BA, McLaughlin JW, Lindo Z. 2015. Climate change drives a shift in peatland ecosystem plant community: Implications for ecosystem function and stability. Global Change Biology 21:388–395. doi: 10.1111/gcb.12643 |
| 1. Dijkstra JA, Westerman EL, Harris LG. 2011. The effects of climate change on species composition, succession and phenology: a case study. Global Change Biology 17:2360–2369. doi: 10.1111/j.1365-2486.2010.02371.x |
| 1. Dijkstra JA, Westerman EL, Harris LG. 2011. The effects of climate change on species composition, succession and phenology: a case study. Global Change Biology 17:2360–2369. doi: 10.1111/j.1365-2486.2010.02371.x |
| 1. Elmendorf SC, Henry GHR, Hollister RD, et al. 2015. Experiment, monitoring, and gradient methods used to infer climate change effects on plant communities yield consistent patterns. Proceedings of the National Academy of Sciences of the United States of America 112:448–452. doi: 10.1073/pnas.1410088112 |
| 1. Engel EC, Weltzin JF, Norby RJ, Classen AT. 2009. Responses of an old-field plant community to interacting factors of elevated CO2 , warming, and soil moisture. Journal of Plant Ecology 2:1–11. doi: 10.1093/jpe/rtn026 |
| 1. Erschbamer B, Unterluggauer P, Winkler E, Mallaun M. 2011. Changes in plant species diversity revealed by long-term monitoring on mountain summits in the Dolomites (northern Italy). Preslia 83:387–401. |
| 1. Farrer EC, Ashton IW, Spasojevic MJ, et al. 2015. Indirect effects of global change accumulate to alter plant diversity but not ecosystem function in alpine tundra. Journal of Ecology 103:351–360. doi: 10.1111/1365-2745.12363 |
| 1. Feeley KJ, Malhi Y, Zelazowski P, Silman MR. 2012. The relative importance of deforestation, precipitation change, and temperature sensitivity in determining the future distributions and diversity of Amazonian plant species. Global Change Biology 18:2636–2647. doi: 10.1111/j.1365-2486.2012.02719.x |
| 1. Felton A, Lindbladh M, Elmberg J, et al. 2014. Projecting impacts of anthropogenic climatic change on the bird communities of southern Swedish spruce monocultures: will the species poor get poorer? Ornis Fennica 91:1–13. |
| 1. Fitzpatrick MC, Sanders NJ, Ferrier S, et al. 2011. Forecasting the future of biodiversity: a test of single- and multi-species models for ants in North America. Ecography 34:836–847. doi: 10.1111/j.1600-0587.2011.06653.x |
| 1. Fonty E, Sarthou C, Larpin D, Ponge J-F. 2009. A 10-year decrease in plant species richness on a neotropical inselberg: detrimental effects of global warming? Global Change Biology 15:2360–2374. doi: 10.1111/j.1365-2486.2009.01923.x |
| 1. Frenette-Dussault C, Shipley B, Meziane D, Hingrat Y. 2013. Trait-based climate change predictions of plant community structure in arid steppes. Journal of Ecology 101:484–492. doi: 10.1111/1365-2745.12040 |
| 1. Garcia-Fayos P, Bochet E. 2009. Indication of antagonistic interaction between climate change and erosion on plant species richness and soil properties in semiarid Mediterranean ecosystems. Global Change Biology 15:306–318. doi: 10.1111/j.1365-2486.2008.01738.x |
| 1. Garcia A, Ortega-Huerta MA, Martinez-Meyer E. 2014. Potential distributional changes and conservation priorities of endemic amphibians in western Mexico as a result of climate change. Environmental Conservation 41:1–12. doi: 10.1017/s0376892913000349 |
| 1. Gaüzère P, Jiguet F, Devictor V. 2015. Rapid adjustment of bird community compositions to local climatic variations and its functional consequences. Global Change Biology 21:3367–3378. doi: 10.1111/gcb.12917 |
| 1. Gedan KB, Bertness MD. 2009. Experimental warming causes rapid loss of plant diversity in New England salt marshes. Ecology Letters 12:842–848. doi: 10.1111/j.1461-0248.2009.01337.x |
| 1. Genner MJ, Sims DW, Southward AJ, et al. 2010. Body size-dependent responses of a marine fish assemblage to climate change and fishing over a century-long scale. Global Change Biology 16:517–527. doi: 10.1111/j.1365-2486.2009.02027.x |
| 1. Gigauri K, Akhalkatsi M, Nakhutsrishvili G, Abdaladze O. 2013. Monitoring of vascular plant diversity in a changing climate in the alpine zone of the Central Greater Caucasus. Turkish Journal of Botany 37:1104–1114. doi: 10.3906/bot-1301-38 |
| 1. Greene C, Kuehne L, Rice C, et al. 2015. Forty years of change in forage fish and jellyfish abundance across greater Puget Sound, Washington (USA): anthropogenic and climate associations. Marine Ecology Progress Series 525:153–170. doi: 10.3354/meps11251 |
| 1. Hájková P, Hájek M, Rybníček K, et al. 2011. Long-term vegetation changes in bogs exposed to high atmospheric deposition, aerial liming and climate fluctuation. Journal of Vegetation Science 22:891–904. doi: 10.1111/j.1654-1103.2011.01297.x |
| 1. Henderson PA, Seaby RMH, Somes JR. 2011. Community level response to climate change: The long-term study of the fish and crustacean community of the Bristol Channel. Journal of Experimental Marine Biology and Ecology 400:78–89. doi: 10.1016/j.jembe.2011.02.028 |
| 1. Heubes J, Schmidt M, Stuch B, et al. 2013. The projected impact of climate and land use change on plant diversity: An example from West Africa. Journal of Arid Environments 96:48–54. doi: 10.1016/j.jaridenv.2013.04.008 |
| 1. Hiddink J, ter Hofstede R. 2008. Climate induced increases in species richness of marine fishes. Global Change Biology 14:453–460. doi: 10.1111/j.1365-2486.2007.01518.x |
| 1. Hiddink JG, Coleby C. 2012. What is the effect of climate change on marine fish biodiversity in an area of low connectivity, the Baltic Sea? Global Ecology and Biogeography 21:637–646. doi: 10.1111/j.1466-8238.2011.00696.x |
| 1. Hiddink JG, Burrows MT, García Molinos J. 2015. Temperature tracking by North Sea benthic invertebrates in response to climate change. Global Change Biology 21:117–129. doi: 10.1111/gcb.12726 |
| 1. Hodd RL, Bourke D, Skeffington MS. 2014. Projected Range Contractions of European Protected Oceanic Montane Plant Communities: Focus on Climate Change Impacts Is Essential for Their Future Conservation. PLoS One. doi: 10.1371/journal.pone.0095147 |
| 1. Horta e Costa B, Assis J, Franco G, et al. 2014. Tropicalization of fish assemblages in temperate biogeographic transition zones. Marine Ecology Progress Series 504:241–252. doi: 10.3354/meps10749 |
| 1. Hudson JMG, Henry GHR. 2009. Increased plant biomass in a High Arctic heath community from 1981 to 2008. Ecology 90:2657–2663. doi: 10.1890/09-0102.1 |
| 1. Hudson JMG, Henry GHR. 2010. High Arctic plant community resists 15 years of experimental warming. Journal of Ecology 98:1035–1041. doi: 10.1111/j.1365-2745.2010.01690.x |
| 1. Huntley B, Collingham YC, Green RE, et al. 2006. Potential impacts of climatic change upon geographical distributions of birds. Ibis 148:8–28. doi: 10.1111/j.1474-919X.2006.00523.x |
| 1. Huntley B, Collingham YC, Willis SG, Green RE. 2008. Potential Impacts of Climatic Change on European Breeding Birds. PLoS One. doi: 10.1371/journal.pone.0001439 |
| 1. Huntley B, Barnard P. 2012. Potential impacts of climatic change on southern African birds of fynbos and grassland biodiversity hotspots. Diversity and Distributions 18:769–781. doi: 10.1111/j.1472-4642.2012.00890.x |
| 1. Jantsch MC, Fischer A, Fischer HS, Winter S. 2013. Shift in Plant Species Composition Reveals Environmental Changes During the Last Decades: A Long-Term Study in Beech (Fagus sylvatica) Forests in Bavaria, Germany. Folia Geobotanica 48:467–491. doi: 10.1007/s12224-012-9148-7 |
| 1. Jetz W, Wilcove DS, Dobson AP. 2007. Projected Impacts of Climate and Land-Use Change on the Global Diversity of Birds. PLoS Biology 5:e157. doi: 10.1371/journal.pbio.0050157 |
| 1. Jin X, Shan X, Li X, et al. 2013. Long-term changes in the fishery ecosystem structure of Laizhou Bay, China. Science China Earth Sciences 56:366–374. doi: 10.1007/s11430-012-4528-7 |
| 1. Jonsdottir IS, Magnusson B, Gudmundsson J, et al. 2005. Variable sensitivity of plant communities in Iceland to experimental warming. Global Change Biology 11:553–563. doi: 10.1111/j.1365-2486.2005.00928.x |
| 1. Kardol P, Campany CE, Souza L, et al. 2010. Climate change effects on plant biomass alter dominance patterns and community evenness in an experimental old-field ecosystem. Global Change Biology 16:2676–2687. doi: 10.1111/j.1365-2486.2010.02162.x |
| 1. Kaschner K, Tittensor DP, Ready J, et al. 2011. Current and Future Patterns of Global Marine Mammal Biodiversity. PLoS ONE 6:e19653. doi: 10.1371/journal.pone.0019653 |
| 1. Keith SA, Newton AC, Morecroft MD, et al. 2009. Taxonomic homogenization of woodland plant communities over 70 years. Proceedings of the Royal Society B-Biological Sciences 276:3539–3544. doi: 10.1098/rspb.2009.0938 |
| 1. Kide SO, Mante C, Dubroca L, et al. 2015. Spatio-Temporal Dynamics of Exploited Groundfish Species Assemblages Faced to Environmental and Fishing Forcings: Insights from the Mauritanian Exclusive Economic Zone. PLoS ONE 10:e0141566–e0141566. doi: 10.1371/journal.pone.0141566 |
| 1. Kissling WD, Field R, Korntheuer H, et al. 2010. Woody plants and the prediction of climate-change impacts on bird diversity. Philosophical Transactions of the Royal Society B-Biological Sciences 365:2035–2045. doi: 10.1098/rstb.2010.0008 |
| 1. Klanderud K, Totland O. 2007. The relative role of dispersal and local interactions for alpine plant community diversity under simulated climate warming. Oikos 116:1279–1288. doi: 10.1111/j.2007.0030-1299.15906.x |
| 1. Koslow JA, Goericke R, Watson W. 2013. Fish assemblages in the Southern California Current: relationships with climate, 1951-2008. Fisheries Oceanography 22:207–219. doi: 10.1111/fog.12018 |
| 1. Kreyling J, Jurasinski G, Grant K, et al. 2011. Winter warming pulses affect the development of planted temperate grassland and dwarf-shrub heath communities. Plant Ecology & Diversity 4:13–21. doi: 10.1080/17550874.2011.558125 |
| 1. La Sorte FA, Lee TM, Wilman H, Jetz W. 2009. Disparities between observed and predicted impacts of climate change on winter bird assemblages. Proceedings of the Royal Society B-Biological Sciences 276:3167–3174. doi: 10.1098/rspb.2009.0162 |
| 1. Lasram FBR, Mouillot D. 2009. Increasing southern invasion enhances congruence between endemic and exotic Mediterranean fish fauna. Biological Invasions 11:697–711. doi: 10.1007/s10530-008-9284-4 |
| 1. Leuven RSEW, Hendriks AJ, Huijbregts MAJ, et al. 2011. Differences in sensitivity of native and exotic fish species to changes in river temperature. Current Zoology 57:852–862. |
| 1. Li F, Kwon Y-S, Bae M-J, et al. 2014. Potential Impacts of Global Warming on the Diversity and Distribution of Stream Insects in South Korea. Conservation Biology 28:498–508. doi: 10.1111/cobi.12219 |
| 1. Lindstrom A, Green M, Paulson G, et al. 2013. Rapid changes in bird community composition at multiple temporal and spatial scales in response to recent climate change. Ecography 36:313–322. doi: 10.1111/j.1600-0587.2012.07799.x |
| 1. Loyola RD, Lemes P, Brum FT, et al. 2014. Clade-specific consequences of climate change to amphibians in Atlantic Forest protected areas. Ecography 37:65–72. doi: 10.1111/j.1600-0587.2013.00396.x |
| 1. Maclean IMD, Hopkins JJ, Bennie J, et al. 2015. Microclimates buffer the responses of plant communities to climate change. Global Ecology and Biogeography 24:1340–1350. doi: 10.1111/geb.12359 |
| 1. Mac Nally R, Bennett AF, Thomson JR, et al. 2009. Collapse of an avifauna: climate change appears to exacerbate habitat loss and degradation. Diversity and Distributions 15:720–730. doi: 10.1111/j.1472-4642.2009.00578.x |
| 1. Maes D, Titeux N, Hortal J, et al. 2010. Predicted insect diversity declines under climate change in an already impoverished region. Journal of Insect Conservation 14:485–498. doi: 10.1007/s10841-010-9277-3 |
| 1. Magurran AE, Dornelas M, Moyes F, et al. 2015. Rapid biotic homogenization of marine fish assemblages. Nature Communications 6:8405. doi: 10.1038/ncomms9405 |
| 1. Martínez-Freiría F, Argaz H, Fahd S, Brito JC. 2013. Climate change is predicted to negatively influence Moroccan endemic reptile richness. Implications for conservation in protected areas. Naturwissenschaften 100:877–889. doi: 10.1007/s00114-013-1088-4 |
| 1. McClean CJ, Lovett JC, Kuper W, et al. 2005. African plant diversity and climate change. Annals of the Missouri Botanical Garden 92:139–152. |
| 1. McDonald KW, McClure CJW, Rolek BW, Hill GE. 2012. Diversity of birds in eastern North America shifts north with global warming. Ecology and Evolution 2:3052–3060. doi: 10.1002/ece3.410 |
| 1. McDonnell TC, Belyazid S, Sullivan TJ, et al. 2014. Modeled subalpine plant community response to climate change and atmospheric nitrogen deposition in Rocky Mountain National Park, USA. Environmental Pollution 187:55–64. doi: 10.1016/j.envpol.2013.12.021 |
| 1. Miranda JR, Mouillot D, Hernandez DF, et al. 2005. Changes in four complementary facets of fish diversity in a tropical coastal lagoon after 18 years: a functional interpretation. Marine Ecology Progress Series 304:1–13. |
| 1. Mokany K, Harwood TD, Williams KJ, Ferrier S. 2012. Dynamic macroecology and the future for biodiversity. Global Change Biology 18:3149–3159. doi: 10.1111/j.1365-2486.2012.02760.x |
| 1. Moor H, Hylander K, Norberg J. 2015. Predicting climate change effects on wetland ecosystem services using species distribution modeling and plant functional traits. Ambio 44:S113–S126. doi: 10.1007/s13280-014-0593-9 |
| 1. Moradi H, Fakheran S, Peintinger M, et al. 2012. Profiteers of environmental change in the Swiss Alps: increase of thermophilous and generalist plants in wetland ecosystems within the last 10 years. Alpine Botany 122:45–56. doi: 10.1007/s00035-012-0102-3 |
| 1. Munson SM, Webb RH, Belnap J, et al. 2012. Forecasting climate change impacts to plant community composition in the Sonoran Desert region. Global Change Biology 18:1083–1095. doi: 10.1111/j.1365-2486.2011.02598.x |
| 1. Nooten SS, Andrew NR, Hughes L. 2014. Potential Impacts of Climate Change on Insect Communities: A Transplant Experiment. PLoS ONE 9:e85987. doi: 10.1371/journal.pone.0085987 |
| 1. Ochoa-Ochoa LM, Rodriguez P, Mora F, et al. 2012. Climate change and amphibian diversity patterns in Mexico. Biological Conservation 150:94–102. doi: 10.1016/j.biocon.2012.03.010 |
| 1. Paesch L, Norbis W, Inchausti P. 2014. Effects of fishing and climate variability on spatio-temporal dynamics of demersal chondrichthyans in the Rio de la Plata, SW Atlantic. Marine Ecology Progress Series 508:187–200. doi: 10.3354/meps10878 |
| 1. Parolo G, Rossi G. 2008. Upward migration of vascular plants following a climate warming trend in the Alps. Basic and Applied Ecology 9:100–107. doi: 10.1016/j.baae.2007.01.005 |
| 1. Penczak T. 2015. Despite anthropogenic disturbance and moderate climate changes fish density and biomass fluctuated non directionally in a small stream. Journal of Limnology 74:286–293. doi: 10.4081/jlimnol.2014.1079 |
| 1. Penuelas J, Prieto P, Beier C, et al. 2007. Response of plant species richness and primary productivity in shrublands along a north-south gradient in Europe to seven years of experimental warming and drought: reductions in primary productivity in the heat and drought year of 2003. Global Change Biology 13:2563–2581. doi: 10.1111/j.1365-2486.2007.01464.x |
| 1. Perez-Rodriguez A, Koen-Alonso M, Saborido-Rey F. 2012. Changes and trends in the demersal fish community of the Flemish Cap, Northwest Atlantic, in the period 1988-2008. Ices Journal of Marine Science 69:902–912. doi: 10.1093/icesjms/fss019 |
| 1. Pletterbauer F, Melcher AH, Ferreira T, Schmutz S. 2015. Impact of climate change on the structure of fish assemblages in European rivers. Hydrobiologia 744:235–254. doi: 10.1007/s10750-014-2079-y |
| 1. Pomati F, Matthews B, Jokela J, et al. 2012. Effects of re-oligotrophication and climate warming on plankton richness and community stability in a deep mesotrophic lake. Oikos 121:1317–1327. doi: 10.1111/j.1600-0706.2011.20055.x |
| 1. Pompe S, Hanspach J, Badeck F, et al. 2008. Climate and land use change impacts on plant distributions in Germany. Biology Letters 4:564–567. doi: 10.1098/rsbl.2008.0231 |
| 1. Pompe S, Hanspach J, Badeck F-W, et al. 2010. Investigating habitat-specific plant species pools under climate change. Basic and Applied Ecology 11:603–611. doi: 10.1016/j.baae.2010.08.007 |
| 1. Prince K, Lorrilliere R, Barbet-Massin M, Jiguet F. 2013. Predicting the fate of French bird communities under agriculture and climate change scenarios. Environmental Science & Policy 33:120–132. doi: 10.1016/j.envsci.2013.04.009 |
| 1. Randin CF, Engler R, Pearman PB, et al. 2010. Using Georeferenced Databases to Assess the Effect of Climate Change on Alpine Plant Species and Diversity. |
| 1. Reif J, Prylová K, Šizling AL, et al. 2013. Changes in bird community composition in the Czech Republic from 1982 to 2004: Increasing biotic homogenization, impacts of warming climate, but no trend in species richness. Journal of Ornithology 154:359–370. doi: 10.1007/s10336-012-0900-9 |
| 1. Reside AE, VanDerWal J, Kutt AS. 2012. Projected changes in distributions of Australian tropical savanna birds under climate change using three dispersal scenarios. Ecology and Evolution 2:705–718. doi: 10.1002/ece3.197 |
| 1. Reu B, Zaehle S, Proulx R, et al. 2011. The role of plant functional trade-offs for biodiversity changes and biome shifts under scenarios of global climatic change. Biogeosciences 8:1255–1266. doi: 10.5194/bg-8-1255-2011 |
| 1. Roth T, Plattner M, Amrhein V. 2014. Plants, Birds and Butterflies: Short-Term Responses of Species Communities to Climate Warming Vary by Taxon and with Altitude. PLoS ONE 9:e82490. doi: 10.1371/journal.pone.0082490 |
| 1. Savage J, Vellend M. 2014. Elevational shifts, biotic homogenization and time lags in vegetation change during 40 years of climate warming. Ecography n/a-n/a. doi: 10.1111/ecog.01131 |
| 1. Schmidt NM, Kristensen DK, Michelsen A, Bay C. 2012. High Arctic plant community responses to a decade of ambient warming. Biodiversity 13:191–199. doi: 10.1080/14888386.2012.712093 |
| 1. Schuch S, Wesche K, Schaefer M. 2012. Long-term decline in the abundance of leafhoppers and planthoppers (Auchenorrhyncha) in Central European protected dry grasslands. Biological Conservation 149:75–83. doi: 10.1016/j.biocon.2012.02.006 |
| 1. Sebastia M-T, Kirwan L, Connolly J. 2008. Strong shifts in plant diversity and vegetation composition in grassland shortly after climatic change. Journal of Vegetation Science 19:299-U27. doi: 10.3170/2008-8-18356 |
| 1. Shackell NL, Bundy A, Nye JA, Link JS. 2012. Common large-scale responses to climate and fishing across Northwest Atlantic ecosystems. Ices Journal of Marine Science 69:151–162. doi: 10.1093/icesjms/fsr195 |
| 1. Shan X, Sun P, Jin X, et al. 2013. Long-Term Changes in Fish Assemblage Structure in the Yellow River Estuary Ecosystem, China. Marine and Coastal Fisheries 5:65–78. doi: 10.1080/19425120.2013.768571 |
| 1. Shi Z, Sherry R, Xu X, et al. 2015. Evidence for long-term shift in plant community composition under decadal experimental warming. Journal of Ecology 103:1131–1140. doi: 10.1111/1365-2745.12449 |
| 1. Simpson SD, Jennings S, Johnson MP, et al. 2011. Continental Shelf-Wide Response of a Fish Assemblage to Rapid Warming of the Sea. Current Biology 21:1565–1570. doi: 10.1016/j.cub.2011.08.016 |
| 1. Sommer JH, Kreft H, Kier G, et al. 2010. Projected impacts of climate change on regional capacities for global plant species richness. Proceedings of the Royal Society B-Biological Sciences 277:2271–2280. doi: 10.1098/rspb.2010.0120 |
| 1. Stralberg D, Jongsomjit D, Howell CA, et al. 2009. Re-Shuffling of Species with Climate Disruption: A No-Analog Future for California Birds? PLoS One. doi: 10.1371/journal.pone.0006825 |
| 1. Telwala Y, Brook BW, Manish K, Pandit MK. 2013. Climate-Induced Elevational Range Shifts and Increase in Plant Species Richness in a Himalayan Biodiversity Epicentre. PLoS One. doi: 10.1371/journal.pone.0057103 |
| 1. ter Hofstede R, Hiddink J, Rijnsdorp A. 2010. Regional warming changes fish species richness in the eastern North Atlantic Ocean. Marine Ecology Progress Series 414:1–9. doi: 10.3354/meps08753 |
| 1. ter Hofstede R, Rijnsdorp AD. 2011. Comparing demersal fish assemblages between periods of contrasting climate and fishing pressure. Ices Journal of Marine Science 68:1189–1198. doi: 10.1093/icesjms/fsr053 |
| 1. Thomsen PF, Jorgensen PS, Bruun HH, et al. 2016. Resource specialists lead local insect community turnover associated with temperature - analysis of an 18-year full-seasonal record of moths and beetles. Journal of Animal Ecology 85:251–261. doi: 10.1111/1365-2656.12452 |
| 1. Thuiller W, Lavorel S, Araujo MB, et al. 2005. Climate change threats to plant diversity in Europe. Proceedings of the National Academy of Sciences of the United States of America 102:8245–8250. doi: 10.1073/pnas.0409902102 |
| 1. Thuiller W, Gueguen M, Georges D, et al. 2014. Are different facets of plant diversity well protected against climate and land cover changes? A test study in the French Alps. Ecography 37:1254–1266. doi: 10.1111/ecog.00670 |
| 1. Thuiller W, Pironon S, Psomas A, et al. 2014. The European functional tree of bird life in the face of global change. Nature communications 5:3118. doi: 10.1038/ncomms4118 |
| 1. Tian YJ, Kidokoro H, Watanabe T. 2006. Long-term changes in the fish community structure from the Tsushima warm current region of the Japan/East Sea with an emphasis on the impacts of fishing and climate regime shift over the last four decades. Progress in Oceanography 68:217–237. doi: 10.1016/j.pocean.2006.02.009 |
| 1. Tisseuil C, Leprieur F, Grenouillet G, et al. 2012. Projected impacts of climate change on spatio-temporal patterns of freshwater fish beta diversity: a deconstructing approach. Global Ecology and Biogeography 21:1213–1222. doi: 10.1111/j.1466-8238.2012.00773.x |
| 1. van der Veer HW, Dapper R, Henderson PA, et al. 2015. Changes over 50 years in fish fauna of a temperate coastal sea: Degradation of trophic structure and nursery function. Estuarine Coastal and Shelf Science 155:156–166. doi: 10.1016/j.ecss.2014.12.041 |
| 1. van Vuuren DP, Sala OE, Pereira HM. 2006. The future of vascular plant diversity under four global scenarios. Ecology and Society 11 [online] |
| 1. Velasquez-Tibata J, Salaman P, Graham CH. 2013. Effects of climate change on species distribution, community structure, and conservation of birds in protected areas in Colombia. Regional Environmental Change 13:235–248. doi: 10.1007/s10113-012-0329-y |
| 1. Villalpando SN, Williams RS, Norby RJ. 2009. Elevated air temperature alters an old-field insect community in a multifactor climate change experiment. Global Change Biology 15:930–942. doi: 10.1111/j.1365-2486.2008.01721.x |
| 1. Virkkala R, Poyry J, Heikkinen RK, et al. 2014. Protected areas alleviate climate change effects on northern bird species of conservation concern. Ecology and Evolution 4:2991–3003. doi: 10.1002/ece3.1162 |
| 1. Walker MD, Wahren CH, Hollister RD, et al. 2006. Plant community responses to experimental warming across the tundra biome. Proceedings of the National Academy of Sciences of the United States of America 103:1342–1346. doi: 10.1073/pnas.0503198103 |
| 1. Walther BA, van Niekerk A. 2015. Effects of climate change on species turnover and body mass frequency distributions of South African bird communities. African Journal of Ecology 53:25–35. doi: 10.1111/aje.12143 |
| 1. White SR, Bork EW, Cahill  Jr. JF. 2014. Direct and indirect drivers of plant diversity responses to climate and clipping across northern temperate grassland. Ecology 95:3093–3103. |
| 1. Yang Y, Wang G, Klanderud K, et al. 2015. Plant community responses to five years of simulated climate warming in an alpine fen of the Qinghai-Tibetan Plateau. Plant Ecology & Diversity 8:211–218. doi: 10.1080/17550874.2013.871654 |
| 1. Yemane D, Mafwila SK, Kathena J, et al. 2015. Spatio-temporal trends in diversity of demersal fish species in the Benguela current large marine ecosystem region. Fisheries Oceanography 24:102–121. doi: 10.1111/fog.12075 |
| 1. Yoshiki TM, Chiba S, Sasaki Y, et al. 2015. Northerly shift of warm-water copepods in the western subarctic North Pacific: Continuous Plankton Recorder samples (2001-2013). Fisheries Oceanography 24:414–429. doi: 10.1111/fog.12119 |
| 1. Zhang M-G, Zhou Z-K, Chen W-Y, et al. 2014. Major declines of woody plant species ranges under climate change in Yunnan, China. Diversity and Distributions 20:405–415. doi: 10.1111/ddi.12165 |
| 1. Zhang Y, Gao Q, Dong S, et al. 2015. Effects of grazing and climate warming on plant diversity, productivity and living state in the alpine rangelands and cultivated grasslands of the Qinghai-Tibetan Plateau. Rangeland Journal 37:57–65. doi: 10.1071/rj14080 |

**General references:**

Albright TP, Pidgeon AM, Rittenhouse CD, et al. 2010. Combined effects of heat waves and droughts on avian communities across the conterminous United States. Ecosphere 1:art12. doi: 10.1890/ES10-00057.1

Albright TP, Pidgeon AM, Rittenhouse CD, et al. 2011. Heat waves measured with MODIS land surface temperature data predict changes in avian community structure. Remote Sensing of Environment 115:245–254. doi: 10.1016/j.rse.2010.08.024

Bailey LD, van de Pol M. 2015. Tackling extremes: Challenges for ecological and evolutionary research on extreme climatic events. Journal of Animal Ecology 85:85–96. doi: 10.1111/1365-2656.12451

Bateman BL, Pidgeon AM, Radeloff VC, et al. 2016. The pace of past climate change vs. potential bird distributions and land use in the United States. Global Change Biology 22:1130–1144. doi: 10.1111/gcb.13154

Bates AE, Barrett NS, Stuart-Smith RD, et al. 2014. Resilience and signatures of tropicalization in protected reef fish communities. Nature Climate Change 4:62–67. doi: 10.1038/nclimate2062

Boucek RE, Rehage JS. 2014. Climate extremes drive changes in functional community structure. Global Change Biology 20:1821–1831. doi: 10.1111/gcb.12574

Butt N, Seabrook L, Maron M, et al. 2015. Cascading effects of climate extremes on vertebrate fauna through changes to low-latitude tree flowering and fruiting phenology. Global Change Biology 21:3267–3277. doi: 10.1111/gcb.12869

Davey CM, Chamberlain DE, Newson SE, et al. 2012. Rise of the generalists: evidence for climate driven homogenization in avian communities. Global Ecology and Biogeography 21:568–578. doi: 10.1111/j.1466-8238.2011.00693.x

Day PD, Stuart-Smith RD, Edgar GJ, Bates AE. Species’ thermal ranges predict changes in reef fish community structure during 8 years of extreme temperature variation.

Devictor V, Julliard R, Couvet D, Jiguet F. 2008. Birds are tracking climate warming, but not fast enough. Proceedings of the ro 275:2743–2748. doi: 10.1098/rspb.2008.0878

Devictor V, van Swaay C, Brereton T, et al. 2012. Differences in the climatic debts of birds and butterflies at a continental scale. Nature Climate Change 2:121–124. doi: 10.1038/nclimate1347

Duque A, Stevenson PR, Feeley KJ. 2015. Thermophilization of adult and juvenile tree communities in the northern tropical Andes. Proceedings of the National Academy of Sciences 112:10744–10749. doi: 10.1073/pnas.1506570112

Garrabou J, Coma R, Bensoussan N, et al. 2009. Mass mortality in Northwestern Mediterranean rocky benthic communities: Effects of the 2003 heat wave. Global Change Biology 15:1090–1103. doi: 10.1111/j.1365-2486.2008.01823.x

Gaüzère P, Jiguet F, Devictor V. 2015. Rapid adjustment of bird community compositions to local climatic variations and its functional consequences. Global Change Biology 21:3367–3378. doi: 10.1111/gcb.12917

Hiddink J, ter Hofstede R. 2008. Climate induced increases in species richness of marine fishes. Global Change Biology 14:453–460. doi: 10.1111/j.1365-2486.2007.01518.x

Jentsch A, Kreyling J, Beierkuhnlein C. 2007. A new generation of climate change experiments : events , not trends. Frontiers in Ecology and the Environment 5:365–374. doi: 10.1890/1540-9295(2007)5[365:ANGOCE]2.0.CO;2

Jiguet F, Julliard R, Thomas CD, et al. 2006. Thermal range predicts bird population resilience to extreme high temperatures. Ecology Letters 9:1321–1330. doi: 10.1111/j.1461-0248.2006.00986.x

Magurran AE, Dornelas M, Moyes F, et al. 2015. Rapid biotic homogenization of marine fish assemblages. Nature Communications 6:8405. doi: 10.1038/ncomms9405

Magurran AE, Henderson PA. 2010. Temporal turnover and the maintenance of diversity in ecological assemblages. Philosophical Transactions of the Royal Society B 365:3611–3620. doi: 10.1098/rstb.2010.0285

Menéndez R, Megías AG, Hill JK, et al. 2006. Species richness changes lag behind climate change. Proceedings of the Royal Society of London Series B: Biological Sciences 273:1465–1470. doi: 10.1098/rspb.2006.3484

Mouthon J, Daufresne M. 2006. Effects of the 2003 heatwave and climatic warming on mollusc communities of the Saone: A large lowland river and of its two main tributaries (France). Global Change Biology 12:441–449. doi: 10.1111/j.1365-2486.2006.01095.x

Parmesan C, Yohe G. 2003. A globally coherent fingerprint of climate change impacts across natural systems. Nature 421:37–42. doi: 10.1038/nature01286

Poloczanska ES, Brown CJ, Sydeman WJ, et al. 2013. Global imprint of climate change on marine life. Nature Climate Change 3:919–925. doi: Doi 10.1038/Nclimate1958

Pratchett MS, Hoey AS, Wilson SK, et al. 2011. Changes in Biodiversity and Functioning of Reef Fish Assemblages following Coral Bleaching and Coral Loss. Diversity 3:424–452.

Roth T, Plattner M, Amrhein V. 2014. Plants, Birds and Butterflies: Short-Term Responses of Species Communities to Climate Warming Vary by Taxon and with Altitude. PLoS ONE 9:e82490. doi: 10.1371/journal.pone.0082490

Sandel B, Arge L, Dalsgaard B, et al. 2011. The Influence of Late Quaternary Climate-Change Velocity on Species Endemism. Science 334:660–664. doi: 10.1126/science.1210173

Seifert LI, Weithoff G, Vos M. 2015. Extreme heat changes post-heat wave community reassembly. Ecology and Evolution 5:2140–2148. doi: 10.1002/ece3.1490

Smale DA, Wernberg T. 2013. Extreme climatic event drives range contraction of a habitat-forming species. Proceedings of the Royal Society B 280:20122829. doi: 10.1098/rspb.2012.2829

Smith MD. 2011. An ecological perspective on extreme climatic events: a synthetic definition and framework to guide future research. Journal of Ecology 99:656–663. doi: 10.1111/j.1365-2745.2011.01798.x

Stralberg D, Jongsomjit D, Howell CA, et al. 2009. Re-Shuffling of Species with Climate Disruption: A No-Analog Future for California Birds? PLoS ONE 4:e6825. doi: 10.1371/journal.pone.0006825

Tayleur CM, Devictor V, Gaüzère P, et al. 2015. Regional variation in climate change winners and losers highlights the rapid loss of cold-dwelling species. Diversity and Distributions 22:1–13. doi: 10.1111/ddi.12412

Thomsen PF, Jorgensen PS, Bruun HH, et al. 2015. Resource specialists lead local insect community turnover associated with temperature - analysis of an 18-year full-seasonal record of moths and beetles. Journal of Animal Ecology 85:251–261. doi: 10.1111/1365-2656.12452

Wernberg T, Bennett S, Babcock RC, et al. 2016. Climate-driven regime shift of a temperature marine ecosystem. Science 353:169–172. doi: 10.1126/science.aad8745

Williams JW, Jackson ST. 2007. Novel climates, no-analog communities, and ecological surprises. Frontiers in Ecology and the Environment 5:475–482. doi: 10.1890/1540-9295(2007)5[475:NCNCAE]2.0.CO;2
